# Evolutionary coincidence of adaptive changes in *exuperantia* and the emergence of *bicoid* in Cyclorrhapha (Diptera)

**DOI:** 10.1007/s00427-017-0594-3

**Published:** 2017-09-11

**Authors:** Janaina Lima de Oliveira, Iderval Silva Sobrinho-Junior, Samira Chahad-Ehlers, Reinaldo Alves de Brito

**Affiliations:** 1 Milner Centre for Evolution, Department of Biology & Biochemistry, University of Bath, Claverton Down, Bath, BA2 7AY UK; 2 Universidade Federal de Goiás, Jataí, 75801-615 Brazil; 3 Departamento de Genética e Evolução, Universidade Federal de São Carlos, São Carlos, 13565-905 Brazil

**Keywords:** *Exuperantia*, Cyclorrhapha, Positive selection, Cooptation, *Bicoid*, *Anastrepha*

## Abstract

The great radiation in the infraorder Cyclorrhapha involved several morphological and molecular changes, including important changes in anterior egg development. During *Drosophila* oogenesis, *exuperantia* (*exu*) is critical for localizing *bicoid* (*bcd*) messenger RNA (mRNA) to the anterior region of the oocyte. Because it is phylogenetically older than *bcd*, which is exclusive to Cyclorrhapha, we hypothesize that *exu* has undergone adaptive changes to enable this new function. Although *exu* has been well studied in *Drosophila*, there is no functional or transcriptional information about it in any other Diptera. Here, we investigate *exu* in the South American fruit fly *Anastrepha fraterculus*, a Cyclorrhapha of great agricultural importance that have lost *bcd*, aiming to understand the evolution of *exu* in this infraorder. We assessed its pattern of gene expression in *A*. *fraterculus* by analyzing transcriptomes from cephalic and reproductive tissues. A combination of next-generation data with classical sequencing procedures enabled identification of the structure of *exu* and its alternative transcripts in this species. In addition to the sex-specific isoforms described for *Drosophila*, we found that not only *exu* is expressed in heads, but this is mediated by two transcripts with a specific 5′UTR exon—likely a result from usage of a third promoter. Furthermore, we tested the hypothesis that *exu* is evolving under positive selection in Cyclorrhapha after divergence from lower Diptera. We found evidence of positive selection at two important exu domains, EXO-like and SAM-like, both involved with mRNA binding during *bcd* mRNA localization in *Drosophila*, which could reflect its cooptation for the new function of *bcd* mRNA localization in Cyclorrhapha.

**Electronic supplementary material:**

The online version of this article (10.1007/s00427-017-0594-3) contains supplementary material, which is available to authorized users.

## Introduction

Cyclorrhapha (Diptera) is a highly diverse group of flies that underwent a major adaptive radiation that led to over 72,000 species (Grimaldi and Engel 2005), which share attributes such as a 360° rotation of the male terminalia and development of a puparium (Yeates and Wiegmann 1999; Yeates et al. 2007). Many molecular studies on Cyclorrhapha have been focused on solving its phylogenetic history (Yeates et al. 2007; Bertone and Wiegmann 2009), though some have investigated molecular changes involved with evolutionary novelties, which identified the acquisition of new functional genes (Stauber et al. 1999; Huchard et al. 2006) and regulatory regions (Lemke et al. 2008).

In *Drosophila*, the products of genes *bicoid* (*bcd*) and *exuperantia* (*exu*) interact with each other and are essential for anterior development, such as the formation of the embryo head and thorax (Schupbach and Wieschaus 1986; Berleth et al. 1988). *bcd* arose from a duplication of *Hox3* in an ancestral of Cyclorrhapha, being unique to this group (Stauber et al. 1999). Key questions in developmental biology are to understand how *bcd* has become the major determinant of anteroposterior patterning, given that a whole new pathway has evolved, and how old components were rearranged in this process (Lynch and Desplan 2003). In this regard, investigations in lower dipteran and other insect orders (therefore, before the emergence of *bcd*) have shown that *ocelliless* (*oc*; previously called *orthodenticle*), *hunchback* (*hb*), and *caudal* (*cad*), also present in Cyclorrhapha, might be the major components of axis determination in these organisms (Wolff et al. 1998; Dearden and Akam 1999; Lynch and Desplan 2003). In higher Diptera, these three genes, among at least 20 others (Ochoa-Espinosa et al. 2005), are under Bcd transcriptional (*oc* and *hb*) or translational (*cad*) regulation (Ephrussi and St Johnston 2004). Interestingly, Bcd-dependent *hunchback* activation, mediated by a Bcd-binding region, is a Cyclorrhapha synapomorphy (Lemke et al. 2008), highlighting how molecular changes allowed *bcd* to become a major regulator in anteroposterior patterning. It has been suggested that changes on how anteroposterior axis is accomplished may have allowed for a faster development, since segmentation occurs at once along the embryo, while there is a delay in this process in more basal lineages of Diptera (McGregor 2005). However, it has also been shown that some cyclorrhaphan flies have lost *bcd* (Klomp et al. 2015), and the mechanism of anteroposterior axis determination remains so far unknown in these species.

Exu associates with microtubules to establish the localization of *bcd* messenger RNAs (mRNAs) to the anterior (Schupbach and Wieschaus 1986; Berleth et al. 1988; Pokrywka and Stephenson 1991; Cha et al. 2001), and *oskar* mRNAs to the posterior (Ephrussi and Lehmann 1992; Wilhelm et al. 2000) oocyte regions during *Drosophila* oogenesis. Besides its function in oogenesis, *exu* is also important in spermatogenesis, given that male *exu* mutants are sterile because sperms fail to develop a tail and do not leave the seminal vesicle (Hazelrigg et al. 1990; Crowley and Hazelrigg 1995). In *D*. *melanogaster*, *exu* expression occurs in the reproductive tissues of both sexes and involves differential splicing of sex-specific isoforms that differ only in the untranslated regions (5′ and 3′UTR), reflecting the use of different promoters and polyadenylation sites (Hazelrigg et al. 1990; Hazelrigg and Tu 1994; Crowley and Hazelrigg 1995). Because the coding region remains the same in both sexes, a single resulting peptide is produced, a pseudonuclease which contains an EXO-like and a SAM-like domains, both implicated in RNA binding during *bcd* mRNA localization (Lazzaretti et al. 2016).

There is evidence indicating that *exu* is phylogenetically older than *bcd*, being observed in several insect orders, even in the most basal, e.g., Orthoptera (Zeng et al. 2013). Since it is only in Cyclorrhapha that *exu* assumes its new role in *bcd* mRNA localization, adaptive changes must have happened that triggered, or allowed, for that change. In order to detect regions in the protein involved with this adaptation in Cyclorrhapha, as well as investigate evolutionary forces shaping them, we studied the evolution of the *exu* gene in Diptera. The South American fruit fly *Anastrepha fraterculus* is a Tephritidae with a wide distribution in South America, which constitutes an important group to understand *exu* evolution in Cyclorrhapha, since the Tephritidae family has lost *bcd* (Klomp et al. 2015). The recently published head transcriptome (Rezende et al. 2016), in conjunction with gene expression data from reproductive tissues (Congrains, C.; Campanini, E.B.; Nakamura, A.M.; de Oliveira, J.L.; Lima, A.L.A.; Chahad-Ehlers, S.; Sobrinho-Junior, I.; de Brito, R.A.: Evidence of sexual selection in reproductive transcriptomes of South American and West Indies fruit flies, *Anastrepha fraterculus* and *Anastrepha obliqua* (Diptera:Tephritidade), unpublished), provide a great opportunity to study *exu* expression in other body structures and species of Diptera. Because it is an important agricultural pest (Zucchi 2000), several strategies have been established for the control of *A*. *fraterculus* (Aluja 1994; Carvalho et al. 2000), particularly with regard to genes involved with reproduction, such as *exu*, which are invaluable as potential targets for silencing and development of sterile insect techniques (SIT) (Knipling 1955).

Aiming to understand both expression and evolutionary patterns of *exu* in Diptera, we characterized this gene in *A*. *fraterculus* and tested the hypothesis that *exu* has undergone positive selection in Cyclorrhapha by investigating its sequence in other Diptera species available on GenBank. We report at least four sites evolving under positive selection in Cyclorrhapha, all of them located in two important Exu domains implicated in RNA binding. These adaptive changes may have occurred in conjunction with other modifications in genes involved in the establishment of the anteroposterior axis, which allowed for the integration of *bicoid* in this process and have led to changes that may have been significant for the evolutionary success of Cyclorrhapha.

## Materials and methods

### Characterization of *exu* isoforms in cephalic and reproductive tissues of *A*. *fraterculus*

Specimens of *A*. *fraterculus* were derived from a population in the Southeastern region of Brazil (São Carlos, 22° 01′ 03″ S, 47° 53′ 27″ W), which has been kept in the laboratory as large stock colonies to prevent genetic drift. Details about the transcriptomes are presented elsewhere (Rezende et al. 2016). Briefly, samples included cephalic and reproductive tissues from mature virgin and post-mating males and females, as well as post-oviposition females. For each of the 10 profiles, a biological replica was also sampled, totaling 20 libraries sequenced on an Illumina HiSeq™ 2000, producing 2 × 100 bp paired-end reads. Reads were first trimmed for quality and length, then assembled using Trinity package (Grabherr et al. 2011) into four major profiles: male and female head tissues, male and female reproductive tissues.

Transcripts were annotated using BLAST tool in the package Standalone BLAST Setup for Unix (Altschul et al. 1990) using Gene Ontology (GO) as reference. Contigs annotated as *exu* were selected for further analysis. In silico inference of the open reading frames (ORFs) and protein sequences (Bikandi et al. 2004) provided initial insights on the transcript structures. Primers were designed for each putative alternative exon to validate these exons and elucidate gene structure (see below). Using cDNA as template, synthesized from a pool of all RNA samples collected for transcriptome sequencing, 3′UTRs were investigated by RACE PCR using FirstChoice® RLM-RACE kit (Ambion). The specific *exu* primers used in amplification reactions were located within the coding region. Amplification of the coding sequence was performed using this same pooled cDNA template, in order to investigate the existence of alternative splicing in this region. PCR products were PEG purified (Lis and Schleif 1975) and cloned using InsTAclone kit (Fermentas). Positive clones were amplified with universal primers M13 (Dallas-Yang et al. 1998), PEG purified and Sanger-sequenced with these same primers on an ABI 3730 sequencer at Macrogen Inc., Korea. Electropherograms were analyzed using Finch TV 1.4.0 (Geospiza, Inc.; Seattle, WA, USA; http://www.geospiza.com) and sequences manually aligned with each other and the *exu* contigs using BioEdit 7.1.3.0 (Hall 1999). Sequences of all *exu* isoforms in *A*. *fraterculus* are available in GenBank under the following accession numbers: KY682091 (female head isoform), KY695250 (male head isoform), KY695249 (female reproductive isoform), and KY695251 (male reproductive isoform).

## Structural organization of *exu* in *A*. *fraterculus*

We extracted DNA of a specimen from the same population described earlier, following a modified protocol from Nelson and Krawetz (1992), and assessed its quality on a 1% agarose gel stained with ethidium bromide. To understand how exons are organized in the gene sequence, we performed different sets of amplification reactions, considering all combinations of forward (f) and reverse (r) primers from alternative 5′ and 3′UTRs. Positive amplifications were identified in agarose gels, purified, cloned, and sequenced as described earlier. High-quality sequences were manually aligned to each other and against *exu* contigs using BioEdit 7.1.3.0 (Hall 1999) in order to identify putative introns and the gene structure sequence of *exu* in *A*. *fraterculus* (GenBank accession number KY695252).

### Hierarchical patterns of molecular variation: Cyclorrhapha vs Diptera

We aligned the *exu* CDS obtained here for the cyclorrhaphan *A*. *fraterculus* to coding sequences of *exu* from 13 other Diptera available on GenBank—six Cyclorrhapha: *Ceratitis capitata* (XM_004526429.1), *Musca domestica* (KA645318.1), *Drosophila melanogaster* (NM_001169747.1), *D*. *mojavensis* (XM_002004251.1), *D*. *virilis* (XM_002059408.1), and *D*. *pseudoobscura* (XM_001360949.2); and seven lower Diptera: *Sitodiplosis mosellana* (GAKJ01009913.1), *Corethrella appendiculata* (GANO01001921.1), *Aedes aegypti* (XM_001654190.1), *Anopheles gambiae* (XM_308467.4), *Phlebotomus papatasi* (JP555152.1), *Chironomus riparius* (KA182541.1), and *Belgica antartica* (GAAK01003495.1). Translated sequences were aligned using MAFFT (Katoh et al. 2002) and the amino acid alignment was manually reverted to the original nucleotide sequence, resulting in a codon-based alignment. The best evolutionary model for *exu* was inferred using jModelTest 2.1.1 (Posada and Crandall 1998; Darriba et al. 2012) considering the Akaike information criterion (AIC). We assessed saturation levels for these sequences plotting the transition and transversion rates versus genetic distances using DAMBE 5.3.74 (Xia 2013). An unrooted maximum likelihood tree of *exu* was inferred using PhyML 3.0 (Guindon and Gascuel 2003; Guindon et al. 2010).

To estimate the pattern of changes in *exu* in Cyclorrhapha and Diptera as a whole, we calculated nonsynonymous/synonymous ratio at two levels: Pi(a)/Pi(s) for Cyclorrhapha and Ka/Ks between this group and lower Diptera. This analysis was carried out in DnaSP version 5 (Librado and Rozas 2009), considering the following parameters: sliding-window and step size of 30 and 3 nucleotides, respectively, discarding gaps. Although Pi(a)/Pi(s) ratio is properly designed for studies of populations rather than species, we performed it here simply as a hierarchical measure of within and between group differences in *exu*.

### Analyses of positive selection

Analyses of positive selection at individual codons were first performed using the MEME test (Murrell et al. 2012) in the webserver Datamonkey (Pond and Frost 2005; Delport et al. 2010). We used mixed effect models, fixed and random effects for sites and branches, respectively, because this test is powerful at identifying episodic diversifying selection. For each codon evolving under positive selection (*p* value < 0.05), posterior probabilities were given to each branch concerning where, within the phylogeny, episodic diversification occurred, and those with *P* > 0.95 were deemed significant. A strict branch-site test (Zhang et al. 2005) on CodeML implemented in the package PAML 4.7 (Yang 2007) was also performed, testing whether sites (codons) in specific branches of the tree were under positive selection (Yang and Nielsen 2002). For this analysis, the phylogenetic tree was divided in two branches: *foreground*, containing Cyclorrhapha species, and *background*, containing lower Diptera. Maximum likelihood estimates for the null model (MA null), in which values of *ω* > 1 are not allowed in either branch, were compared with those of the alternative model (MA), which allows values of *ω* > 1 only in the *foreground* branch, using a likelihood ratio test (LRT; chi-squared null distribution, one degree of freedom) (Yang 2007). Finally, Bayes Empirical Bayes (BEB) was employed to provide posterior probabilities for each codon to be evolving under every evolutionary class (purifying selection: *ω* < 1; neutral: *ω* = 1; positive selection: *ω* > 1) (Yang et al. 2005). Only sites with a posterior probability *P* > 0.95 were deemed significant.

### Analysis of relaxed selection in Tephritidae

Because Tephritidae flies have lost *bcd* (Klomp et al. 2015), we tested the hypothesis that *exu* evolves under relaxed selection in this group, since it no longer performs the function of *bcd* mRNA localization. The test was performed using RELAX (Wertheim et al. 2015), implemented in the webserver Datamonkey (Pond and Frost 2005; Delport et al. 2010). RELAX requires an a priori hypothesis regarding the lineages evolving under relaxed selection and compares the proportion of codons evolving under selection (*ω*) in this group to reference branches. As we are interested in signs of relaxed selection during Tephritidae diversification within Cyclorrhapha, the reference branch in this analysis concerns non-Tephritidae cyclorrhaphan flies (*M*. *domestica*, *D*. *pseudoobscura*, *D*. *melanogaster*, *D*. *mojavensis*, and *D*. *virilis*), while the test branch is formed by Tephritidae species (*A*. *fraterculus* and *C*. *capitata*). The selection intensity parameter *k* is fixed to 1 in both groups in the null model, which is compared to an alternative model where *k* is free to vary in both tests and reference branches by a likelihood ratio test.

## Results and discussion

### *exu* expression is mediated by tissue- and sex-specific isoforms in *A*. *fraterculus*

**Fig. 1:**
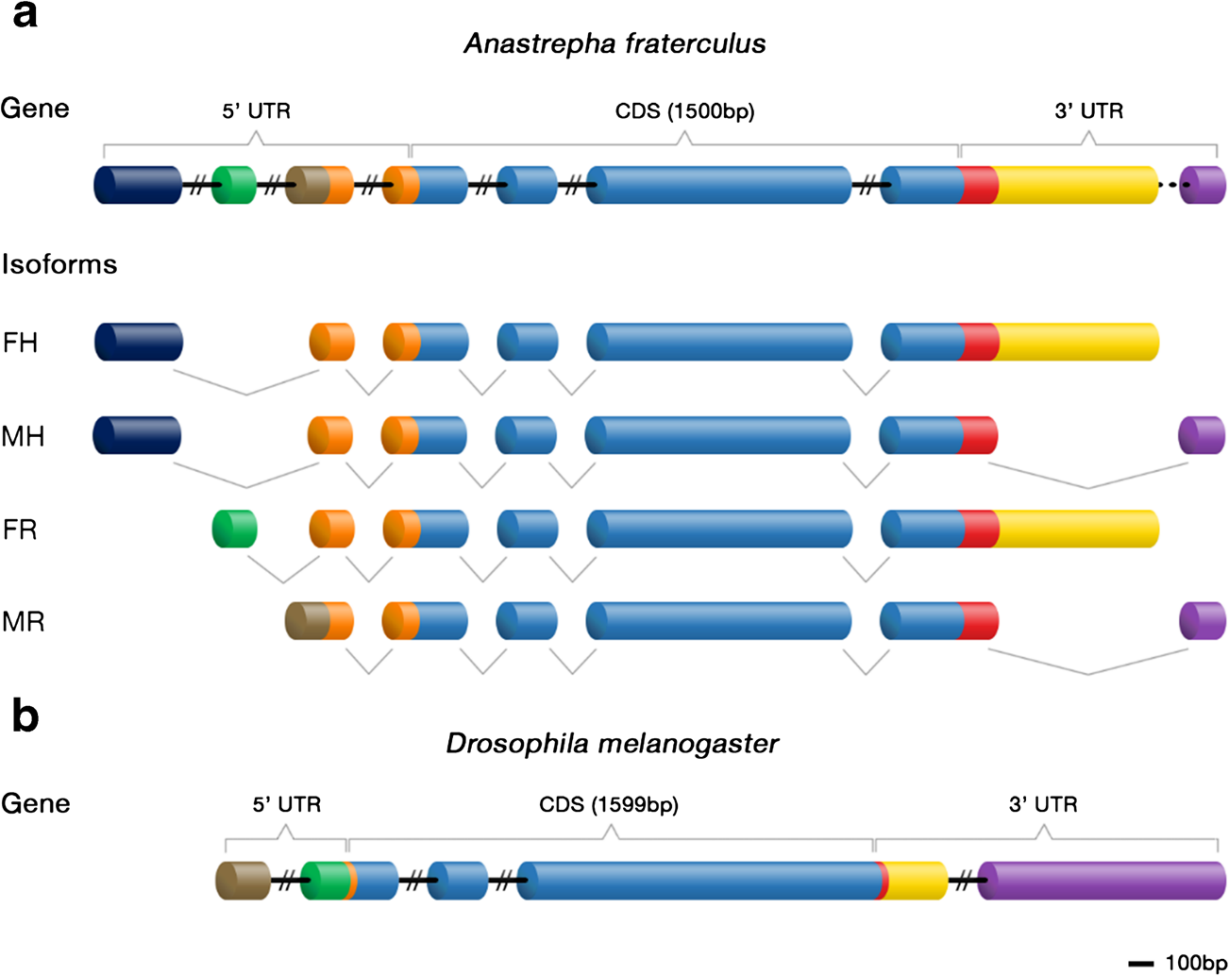
Structure of *exu* gene and isoforms in *A*. *fraterculus* and in *D*. *melanogaster*. **a** Differences among the four isoforms of *exu* in *A*. *fraterculus* are found only at the untranslated regions (UTRs). Female and male head transcripts (FH and MH, respectively) share the same 5′UTR, but have sex-specific 3′UTR exons. In reproductive tissues, besides these sex-specific exons at 3′UTR, female and male isoforms (FR and MR) also differ at 5′UTR. Introns are represented by continuous (confirmed) and dashed (predicted) lines. Only exons are represented proportionally to the reference bar (100 bp), not introns. **b** Structure of *exu* in *D*. *melanogaster*

Using transcriptome data from head (Rezende et al. 2016) and reproductive tissues (Congrains, C.; Campanini, E.B.; Nakamura, A.M.; de Oliveira, J.L.; Lima, A.L.A.; Chahad-Ehlers, S.; Sobrinho-Junior, I.; de Brito, R.A.: Evidence of sexual selection in reproductive transcriptomes of South American and West Indies fruit flies, *Anastrepha fraterculus* and *Anastrepha obliqua* (Diptera:Tephritidade), unpublished), we identified a total of four *exu* isoforms. All *exu* transcripts share the same coding sequence (CDS), though they show a diverse combination of untranslated exons. Transcripts from reproductive tissues differ at both 5′ and 3′UTRs, following the pattern described in *Drosophila* (Hazelrigg et al. 1990; Hazelrigg and Tu 1994; Crowley and Hazelrigg 1995). On the other hand, expression in heads involves two transcripts that share the same 5′UTR, which is not detected in the two reproductive isoforms, but differs at the 3′UTR in a sex-specific manner. Thus, *exu* expression in *A*. *fraterculus* seems to follow the use of three different promoters that depend on tissue and sex, while the polyadenylation sites and alternative splicing of 3′UTR exons are sex-specific only, independent of the tissue (Fig. 1a).

The combination of next-generation sequencing, PCR amplification, and Sanger sequencing allowed us to establish that the *exu* exon organization into the gene structure in *A*. *fraterculus* (Fig. 1a; Supplementary Figure S1) is different when compared to that of *Drosophila* species (Fig. 1b). Besides the 5′UTR exon specific to *Anastrepha* head transcripts, which is located further upstream in the gene, male and female reproductive exons are inverted in *A*. *fraterculus* when compared to those of *D*. *melanogaster* at both 5′ and 3′ untranslated regions. *A*. *fraterculus* also differs in having extra introns not only separating an exon at the 5′UTRs, but also in the coding sequence, since in *A*. *fraterculus*, the CDS is formed by four exons, instead of the three found in *D*. *melanogaster*.

*D*. *melanogaster* was the only insect for which the expression and isoforms of *exu* had been studied in detail. Although four different transcripts were predicted for this species, only two have been experimentally validated (FlyBase Dmel\exu Attrill et al. 2016). These transcripts are sex-specific and expressed in reproductive tissues, being a result of the use of different promoters, polyadenylation sites, and alternative splicing (Hazelrigg and Tu 1994; Crowley and Hazelrigg 1995). We observed a similar pattern in reproductive tissues of *A*. *fraterculus*, in which males and females have specific *exu* transcripts, and the differences are restricted to 5′ and 3′ untranslated regions. Frequently, UTRs contain elements involved in post-transcriptional regulation that determine protein levels in the cell (Chatterjee and Pal 2009). In fact, the male-specific 3′UTR exon of *exu* in *D*. *melanogaster* influences transcript stability and is essential for its role in spermatogenesis (Crowley and Hazelrigg 1995).

There is also evidence of *exu* being expressed, though at very low levels, in somatic tissues of *D*. *melanogaster*, involving an isoform of intermediate size to the reproductive ones (Hazelrigg et al. 1990). Though we have only investigated cephalic and reproductive tissues, our results indicate that there is somatic expression in the cephalic region in the South American fruit fly. This is mediated by two isoforms, which are sex-specific but share the same 5′UTR exclusive to cephalic transcripts, suggesting that *exu* expression may be regulated by a third promoter in this tissue. The 3′ untranslated regions found in both cephalic and reproductive tissues highlight the role of alternative splicing on controlling *exu* expression between sexes, as described for *D*. *melanogaster* (Hazelrigg and Tu 1994; Crowley and Hazelrigg 1995).

The main difference in the coding sequence of *exu* in *A*. *fraterculus*, aside from the extra intron separating its last two exons, is the fact that the predicted protein in this species is shorter, with 499 amino acids, than the 532 residues observed in *D*. *melanogaster*, but it still contains both the EXO-like and SAM-like domains identified in the latter (Moser et al. 1997; Lazzaretti et al. 2016). Interestingly, this final region that corresponds to the fourth exon in *A*. *fraterculus* is only poorly aligned among Diptera species (see below; also mentioned by Lazzaretti et al. 2016). In vivo assays show that truncated mutant alleles lacking this final region of the protein still recover Bcd anterior localization, though their impact on spermatogenesis was not investigated (Lazzaretti et al. 2016). Taken together, these results may reflect a progressive loss due to relaxed or positive selection in the C-terminal region of Exu.

Understanding *exu* structural and expression patterns also has practical implications. *A*. *fraterculus* is an important agricultural pest, since this species colonize a wide range of plant species and the larvae develop inside their fruits (Zucchi 2000). Among several strategies to control this species (Aluja 1994; Carvalho et al. 2000) is the sterile insect technique (SIT) (Knipling 1955), which consists in the liberation of sterile insects in nature. However, these mutants must be equally competitive to the individuals in natural populations, so mutations must affect not their ability to copulate, but to leave a viable offspring. In this regard, we provide information that makes *exu* an interesting target for application of SI techniques.

### Adaptive changes in Cyclorrhapha *exu* within RNA-binding domains

**Fig. 2:**
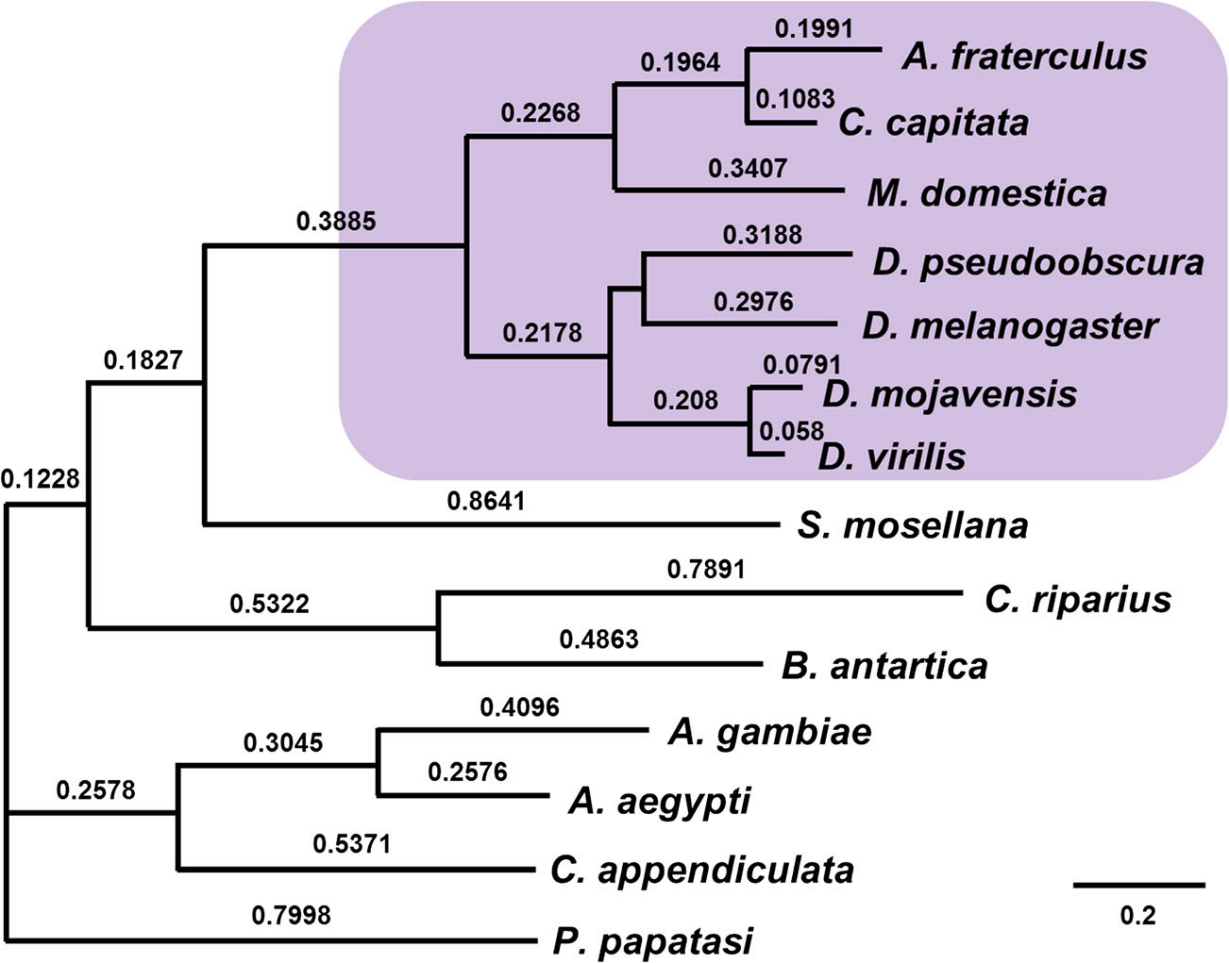
Maximum likelihood unrooted tree of *exu* in Diptera. The *foreground* branch (Cyclorrhapha) is highlighted. Above each branch is the number of nucleotide substitutions per nucleotide site

**Fig. 3:**
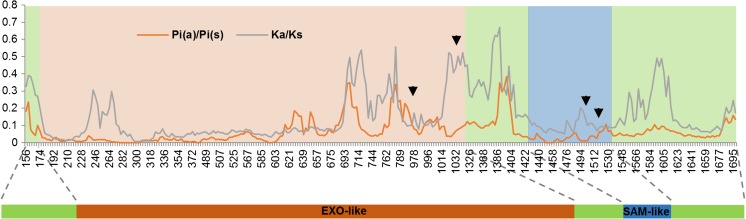
Genetic variation and divergence levels in Cyclorrhapha and Diptera. Estimates of nonsynonymous/synonymous substitutions at two hierarchical levels: within Cyclorrhapha (Pi(a)/Pi(s)) and among this group and lower Diptera (Ka/Ks), considering a sliding-window of 30 nucleotides and a step-size of 3. Gaps were not considered in this analysis. Arrows indicate codons identified as evolving under positive selection considering BEB posterior probabilities

To investigate the evolution of *exu* in Cyclorrhapha, we aligned *exu* sequences from 14 species of Diptera: seven cyclorrhaphan (including *D*. *melanogaster* and *A*. *fraterculus*) and seven lower dipteran species. Further analyses were restricted to the initial 571 codons of the alignment (which corresponds to 411 in *A*. *fraterculus*) due to great divergence at the end of the protein that prevented an adequate amino acid alignment (Supplementary Figure S2; Fig. 2). Importantly, we verified that transition and transversion rates increased continuously with the genetic distance of the investigated species, discarding any possible bias at nonsynonymous to synonymous ratios due to underestimation of synonymous substitutions (Supplementary Figure S3). A hierarchical sliding-window analysis comparing nonsynonymous to synonymous ratios revealed that most variation at this gene is distributed between Cyclorrhapha and lower Diptera—not within Cyclorrhapha (Fig. 3). Interestingly, regions of great divergence are also found within the EXO-like domain (Fig. 3).

By modeling dN/dS at individual sites and allowing each branch to evolve under different evolutionary forces, we were able to provide a more detailed scenario of the evolutionary forces acting on *exu* during the evolution of higher Brachycera. Using such strategy, MEME identified four codons under positive selection in diverse lineages of Diptera, but only two of them involved the Cyclorrhapha branch (Supplementary Fig. S4). Codon 321 (numbered with regard to the total alignment used here, which corresponds to positions 253 in *A*. *fraterculus* and 254 in *D*. *melanogaster*) is located within the EXO-like domain (Moser et al. 1997; Lazzaretti et al. 2016), and the direction of change is towards the fixation of nonpolar (glycin) or positively charged (arginine/histidine) amino acids. Codon 500 (positions 340 in *A*. *fraterculus* and 341 in *D*. *melanogaster*) lies within the SAM-like domain (Lazzaretti et al. 2016), and the substitution is to an arginine, a positively charged amino acid (Supplementay Fig. S2; Fig. 3).

To identify adaptive changes specific to Cyclorrhapha, we set this group as a *foreground* branch and performed a branch-site test, using the more basal species of Diptera as *background* (Fig. 2). Bayes Empirical Bayes (BEB) posterior probabilities identified two sites evolving under positive selection (*P* > 0.95) (Fig. 3; Supplementary Fig. S5), one of which codon 343 (positions 275 in *A*. *fraterculus* and 276 in *D*. *melanogaster*), located within the EXO-like domain, which segregates two amino acids in lower Diptera: alanine (nonpolar) and serine (polar), whereas the residue is always polar and uncharged in Cyclorrhapha, conserving the amino acid physicochemical properties. The second codon under positive selection was in position 506 (positions 346 in *A*. *fraterculus* and 347 in *D*. *melanogaster*), also within the putative SAM-like domain, in which we find only arginine (positively charged) in lower Diptera, while only isoleucine (nonpolar) is observed in Cyclorrhaphan species (Supplementary Fig. S2).

**Fig. 4:**
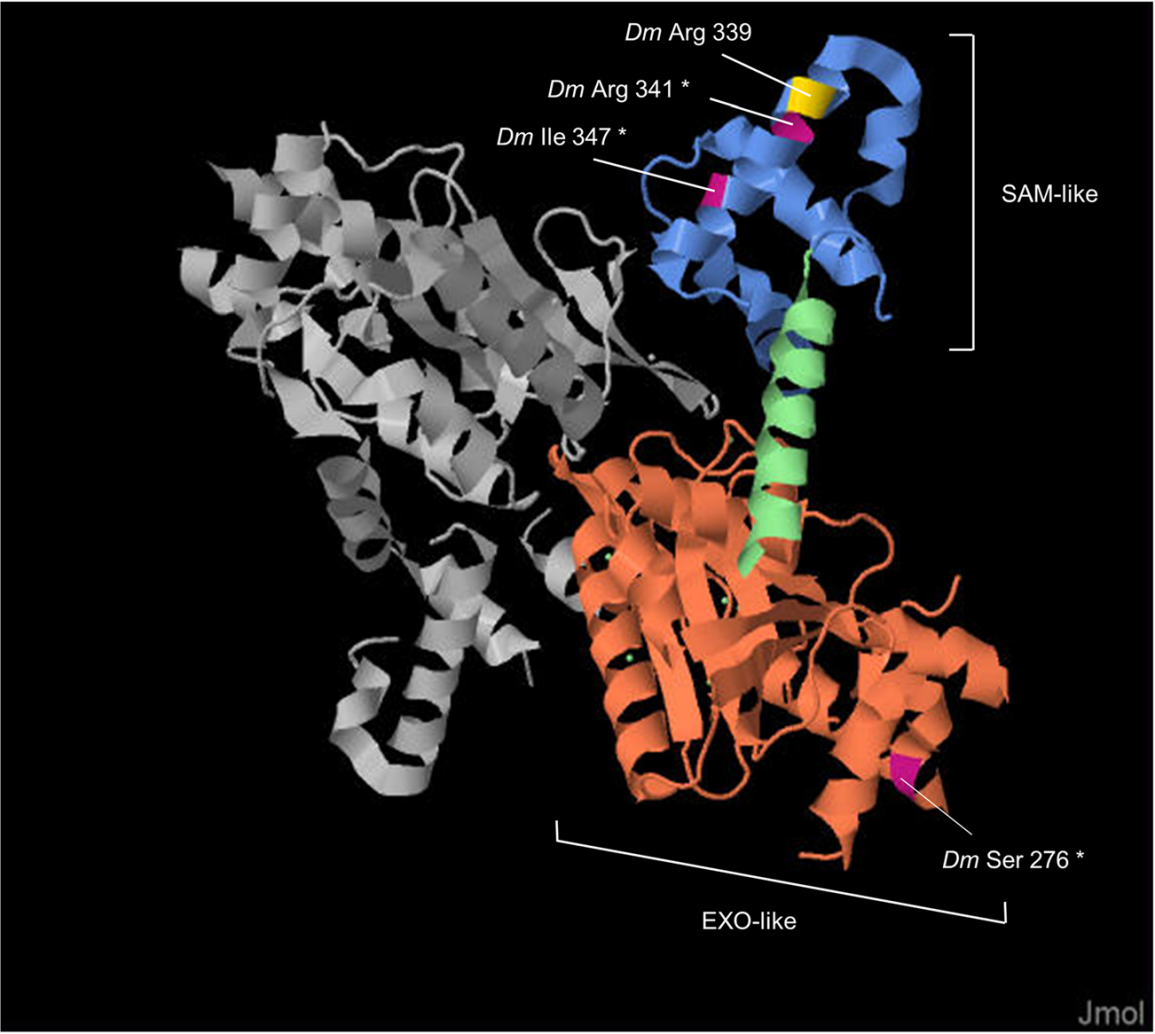
Exu tertiary structure: domains and residues under positive selection. Exu acts as a homodimer. Each monomer contains an EXO-like and a SAM-like domain, connected by a linker (although the two monomers are represented, coordinates are given for only one of them). Among the four sites evolving under positive selection in Cyclorrhapha, three are depicted here (highlighted by asterisks; all residues are numbered according to the *D*. *melanogaster exu* sequence) and form complex secondary structures. Two of them that are in the SAM-like domain are part of the same α-helix as the important Dmel Arg 339

All sites under positive selection in *exu* are located imbedded in regions that BEB found to be under purifying selection (Supplementary Fig. S5), suggesting they are functionally important (Nielsen 2005). The region that contains the first codon under positive selection (321) is not included in the tertiary structure of Exu (Lazzaretti et al. 2016), whereas the remaining three codons (343, 500, and 506) lie within α-helices (Fig. 4). The first two codons are located in an EXO-like domain which it is thought to be a RNA binding site (Marcey et al. 1991; Lazzaretti et al. 2016), whereas the latter two are part of the same α-helix in a SAM-like domain, which also includes an arginine residue that when mutated, disrupts the function of *bcd* mRNA localization in *D*. *melanogaster* (Marcey et al. 1991; Lazzaretti et al. 2016).

### Adaptive changes in *exuperantia* coincide with the establishment of *bicoid* as a major anteroposterior axis regulator in Cyclorrhapha

Exu contains an EXO-like domain derived from a DEDD exonuclease (Moser et al. 1997), but the catalytic activity was lost during arthropod evolution, remaining as an RNA binding region (Lazzaretti et al. 2016). Exu also acquired a SAM-like domain in arthropods, also implicated in RNA binding, and a loop to function as a homodimer. This Exu structure is conserved to all insect lineages, suggesting that it may have a broader function in RNA expression regulation (Lazzaretti et al. 2016). As *bicoid* emerged only in Cyclorrhapha, the ancient function of *exu* could be related to the localization of *oskar* in oocytes (Ephrussi and Lehmann 1992; Wilhelm et al. 2000), spermatogenesis (Hazelrigg et al. 1990), or, based on our results, a role in somatic tissues.

Our findings show that the expression of *exu* in heads is mediated by specific transcripts (different from those detected in reproductive tissues), suggesting the use of a third promoter. There is increasing evidence supporting that the evolution of developmental novelties may arise from changes on regulatory networks and regions (Prud’homme et al. 2007). Whether the different promoters used for *exu* transcription result from its cooption to expression in different sites or sexes, remains an open and exciting question to be investigated. Intriguingly, *oskar*, a gene involved with axial patterning and responsible for assembling the germ pole in *Drosophila* (Ephrussi and Lehmann 1992), was coopted from the neural system before the diversification of holometabolous insects (Lynch et al. 2011; Ewen-Campen et al. 2012). During the process of cooptation, the capacity of being localized at the posterior pole was crucial (Lynch et al. 2011), and, at least in *Drosophila*, this is performed by *exu* (Ephrussi and Lehmann 1992; Wilhelm et al. 2000). Although it is tempting to speculate that *exu* evolution may have followed a similar path—since it is expressed in cephalic tissues, has a specific promoter for reproductive female transcription, and participates on *oskar* localization during oogenesis—this must be investigated considering a large-scale phylogenetic approach regarding structural and expression features of the different isoforms.

Along with *hunchback*, *nanos*, and *caudal*, *bicoid* plays a central role on anteroposterior axis determination, specifically regulating the expression of anterior structures (Gilbert 2000) in *Drosophila*. However, *bcd* has emerged and assumed this important position just before Cyclorrhapha diversification (Stauber et al. 1999, 2002). Although this fact has been considered a great evolutionary breakthrough for providing a faster development (McGregor 2005), the mechanism by which it occurred is poorly understood. We hypothesized that genes that are evolutionarily older than *bcd*, but interact with it in cyclorrhaphan species, may have undergone changes that enabled Bcd integration into the process. Accordingly, the regulatory region of *hunchback* (*hb*) under regulation by Bcd is exclusive to Cyclorrhapha, and must have been acquired alongside *bcd* (Lemke et al. 2008). Thus, we tested the hypothesis that *exu* has undergone adaptive changes that allowed its new role on *bcd* mRNA localization at the anterior pole of the oocyte in Cyclorrhapha.

The first evidence arose from results given by a sliding-window analysis showing that divergence levels between Cyclorrhapha and lower Diptera are higher than those of within the former group (Fig. 3), which is consistent with what is expected under positive selection in a particular group—decreased nonsynonymous to synonymous ratio within this specific lineage and increased divergence between groups (McDonald and Kreitman 1991). Importantly, the analysis identified that this pattern is driven also by regions located within the EXO-like and SAM-like domains.

However, divergence can also be driven by fixation of neutral (Kimura 1968; Kimura and Ohta 1971) or nearly neutral variants under weak selection (Ohta 1992; Akashi et al. 2012) rather than by positive selection. To disentangle these different scenarios, we performed evolutionary tests to specifically identify sites under positive selection. These analyses pointed at four adaptive changes in the Cyclorrhapha lineage, which not only are located in the two most important domains of Exu, but within each domain they are surrounded by sites evolving under strong purifying selection (Supplementary Fig. S5). Regions under purifying selection are usually of functional importance (Nielsen 2005), and given that single nonsynonymous substitutions can have a large effect on an organism or species (Ng and Henikoff 2006), fixation of new mutations in these regions should provide an advantage to be retained by selection. This is even more relevant when we consider that pleiotropy can impose evolutionary constrains to optimal adaptation of individual traits, which is particularly true for genes implicated in early aspects of development, since they are more likely to regulate the expression of a large number of genes (Artieri et al. 2009). Consistent with this idea, a single mutation in the SAM-like domain, specifically in the same α-helix where two out of the four sites under positive selection are located, is sufficient to disrupt *bcd* localization (Marcey et al. 1991; Lazzaretti et al. 2016), indicating that this is a crucial region for mRNA binding.

Adaptation and cooptation of a gene for new molecular functions are not necessarily accomplished by multiple radical changes—indeed, it is often driven by mutations of moderate effect (Fay et al. 2001). Further studies are necessary to assess the exact effect of these changes in Exu, however, considering that they occurred in this specific lineage and in important regions for RNA binding, we suggest that they should be involved with the cooptation of Exu to *bcd* mRNA localization in the Cyclorrhapha lineage.

### *exu* evolution after *bcd* loss in some cyclorrhaphan families

It has been recently demonstrated that at least two cyclorrhaphan families—Tephritidae and Glossinidae—have lost *bcd* (Klomp et al. 2015), which provides an opportunity to test how *exu* evolves under a scenario of secondary loss of *bcd*. We confirmed that this gene is absent in *A*. *fraterculus* (Congrains et al., unpublished) and tested whether after *bcd* loss in Tephritidae (*A*. *fraterculus* + *C*. *capitata* branch in Fig. 2), *exu* evolves under relaxed selection, since it no longer performs the function of *bcd* localization in this group. Surprisingly, not only RELAX failed to identify signs of selection relaxation (or intensification) in this group (Table S1), but we also found that the four sites under positive selection during Cyclorrhapha diversification remain conserved in these two species (Supplementary Fig. S2). Taken together, these findings suggest that once the four minor changes were fixed by positive selection, they remained important to Exu function even after *bcd* loss. Whether the reason is because *exu* continues to be important on localizing a new mRNA molecule that replaces *bcd* in these fruit flies will remain unknown until the mechanism of anteroposterior axis determination is fully understood in Tephritidae. If this is the case, it will be interesting to test whether or not this new cooptation of *exu* in this family was addressed by positive selection in future work, as seems to be the case in Cyclorrhapha.

## Conclusions

We characterized *exuperantia* in the South American fruit fly *A*. *fraterculus* by describing its structure and pattern of expression, expanding the knowledge about this gene beyond the *Drosophila* genus. Besides gonads, *exu* transcripts are also found in cephalic tissues, explaining the somatic expression observed in *Drosophila* (Hazelrigg et al. 1990). This is mediated by two unique isoforms which differ from the ones found in reproductive tissues and are likely to result from the use of a third promoter. The differential splicing and tissue-specific expression makes *exu* an interesting candidate gene for silencing strategy in sterile insect (SI) protocols, since it may be possible to inhibit the translation of specific isoforms in one tissue without affecting its expression in another tissue. Furthermore, we tested the hypothesis that *exu* has undergone adaptive changes in the Cyclorrhapha lineage, involved with its cooption to *bicoid* mRNA localization in this group. This was supported by the finding of four sites under positive selection in the two RNA-binding domains necessary for *bcd* localization. We also suggest that this may be a general pattern to genes that interact up- or downstream with *bcd*.

## Electronic supplementary material

ESM 1 (PDF 691 kb).
