# Supplementary material for: Evolutionary coincidence of adaptive changes in exuperantia and the emergence of bicoid in Cyclorrhapha (Diptera)
Source: Dev Genes Evol. 2017 Sep 11;227(5):355–65. doi: 10.1007/s00427-017-0594-3 (PMC5597691; doi:10.1007/s00427-017-0594-3)
Supplement: Supplementary file 1 — (PDF 691 kb). [file 427_2017_594_MOESM1_ESM.pdf]

Evolutionary coincidence of adaptive changes in *exuperantia* and the emergence of *bicoid* in Cyclorrhapha (Diptera)

Journal: Development Genes and Evolution

Janaina Lima de Oliveira[1*], Iderval Silva Sobrinho-Junior[2], Samira Chahad-Ehlers[3], Reinaldo Alves de Brito[3]

[1]Milner Centre for Evolution, Department of Biology & Biochemistry, University of Bath, BA2 7AY Bath, United Kingdom. [2]Universidade Federal de Goiás, 75801-615 Jataí, Brazil. [3]Departamento de Genética e Evolução, Universidade Federal de São Carlos, 13565-905 São Carlos, Brazil

[*]Correspondence: Janaina Lima de Oliveira, University of Bath. Milner Centre for Evolution, Department of Biology and Biochemistry, Claverton Down, BA2 7AY Bath, United Kingdom. Phone: (+44) 07497524146. E-mail: janajpb2@gmail.com

```
          ....|....|....|....|....|....|....|....|....|....|....|....|....|....|....|....|....|....|....|....|....|....|....|....|....|....|....|....|....|....|
             5        15        25        35        45        55        65        75        85        95       105       115       125       135       145
exu_Gene  CAGCCGTTTCTAGAATATGTAAAGTTTCTTGTTAAACTTTTAAGGTTTTTGGTTATTGCATCGTGGCCCAGAAAACAGAAGTGCATTCAACGAAATATTGCATTTAAAATTTATTTTTTTGAAATTCACACAACTTAGAACAAAAGACTT
exu_FH    CAGCCGTTTCTAGAATATGTAAAGTTTCTTGTTAAACTTTTAAGGTTTTTGGTTATTGCATCGTGGCCCAGAAAACAGAAGTGCATTCAACGAAATATTGCATTTAAAATTTATTTTTTTGAAATTCACACAACTTAGAACAAAAGACTT
exu_MH    CAGCCGTTTCTAGAATATGTAAAGTTTCTTGTTAAACTTTTAAGGTTTTTGGTTATTGCATCGTGGCCCAGAAAACAGAAGTGCATTCAACGAAATATTGCATTTAAAATTTATTTTTTTGAAATTCACACAACTTAGAACAAAAGACTT
exu_FR    ------------------------------------------------------------------------------------------------------------------------------------------------------
exu_MR    ------------------------------------------------------------------------------------------------------------------------------------------------------

          ....|....|....|....|....|....|....|....|....|....|....|....|....|....|....|....|....|....|....|....|....|....|....|....|....|....|....|....|....|....|
               155       165       175       185       195       205       215       225       235       245       255       265       275       285       295
exu_Gene  GCAATATTTAGCTATCCATTAGATCCAAGTGCATATGAAATAATTTGAATGTAAATGATCTTCACTTTGGCCTTAACCTAGTAGTGTAAAATTCTTGGTGATTAAGACTTGGTAAGATCGCGTGACGATGTTGTTGTTGTAACAGCATAG
exu_FH    GCAATATTTAGCTATCCATTAGATCCAAGTGCATATGAAATAATTTGAATGTAAATGATCTTCACTTTGGCCTTAACCTAGTAGTGTAAAATTCTTGGTGATTAAGACTTG---------------------------------------
exu_MH    GCAATATTTAGCTATCCATTAGATCCAAGTGCATATGAAATAATTTGAATGTAAATGATCTTCACTTTGGCCTTAACCTAGTAGTGTAAAATTCTTGGTGATTAAGACTTG---------------------------------------
exu_FR    ------------------------------------------------------------------------------------------------------------------------------------------------------
exu_MR    ------------------------------------------------------------------------------------------------------------------------------------------------------

          ....|....|....|....|....|....|....|....|....|....|....|....|....|....|....|....|....|....|....|....|....|....|....|....|....|....|....|....|....|....|
               305       315       325       335       345       355       365       375       385       395       405       415       425       435       445
exu_Gene  ACATTCCCCCTAAATGTTTGGGAAAAGCGGAGTGACAGAATGGAGTGACATTTTTTAATCGAATATAAATCCGGGTAGTTCTGGTAGCGCAGAACCGACTGTGGTGCGACGTCGCGAAAAGAGCTGTGTGATAAGGCAACTGAGGGATGC
exu_FH    ------------------------------------------------------------------------------------------------------------------------------------------------------
exu_MH    ------------------------------------------------------------------------------------------------------------------------------------------------------
exu_FR    ------------------------------------------------------------------------------------------------------------------------------------------------------
exu_MR    ------------------------------------------------------------------------------------------------------------------------------------------------------

          ....|....|....|....|....|....|....|....|....|....|....|....|....|....|....|....|....|....|....|....|....|....|....|....|....|....|....|....|....|....|
               455       465       475       485       495       505       515       525       535       545       555       565       575       585       595
exu_Gene  GTCTGTAGAAAGGGCAATTTACAATGGTTTTGCCTCACTACTCGCTAAAAAACAATCCAATGCGCGTAGCGTACAATTTCATGTTAAAAAGTTGGCTTGCTTAGCAGATTATTAACGGAAAGTACTTGCAATTCTAGGCACTCATAAATG
exu_FH    ------------------------------------------------------------------------------------------------------------------------------------------------------
exu_MH    ------------------------------------------------------------------------------------------------------------------------------------------------------
exu_FR    ------------------------------------------------------------------------------------------------------------------------------------------------------
exu_MR    ------------------------------------------------------------------------------------------------------------------------------------------------------
```

```
           ....|....|....|....|....|....|....|....|....|....|....|....|....|....|....|....|....|....|....|....|....|....|....|....|....|....|....|....|....|....|
               605       615       625       635       645       655       665       675       685       695       705       715       725       735       745
exu_Gene   ATAATTGAAATTTCGATCGAATACCACCCCAGCCCAGCAGGCGTCATTCGATTGCTCTGATTTTCCGCACAAATTTTCTTTACAGTTGTAACGAAATACATACATACACACATACATGTACATACGTACATAAATACGGTAAAGATCGGC
exu_FH     ------------------------------------------------------------------------------------------------------------------------------------------------------
exu_MH     ------------------------------------------------------------------------------------------------------------------------------------------------------
exu_FR     ------------------------------------------------------------------------------------------------------------------------------------------------------
exu_MR     ------------------------------------------------------------------------------------------------------------------------------------------------------

           ....|....|....|....|....|....|....|....|....|....|....|....|....|....|....|....|....|....|....|....|....|....|....|....|....|....|....|....|....|....|
               755       765       775       785       795       805       815       825       835       845       855       865       875       885       895
exu_Gene   GATCATTTGGTTTTTGTTTTTCGTGCGAATTATTTCAATTCTTGGTGCGCTTTTGCCTTCAGTCCAGAGTGCTGCTTTGCTTTCGGATGTCCACCTGTCAACGATCAATACACGGCTTGAAATTGTTTGTATCTAAACTGTGAGAGAAGG
exu_FH     ------------------------------------------------------------------------------------------------------------------------------------------------------
exu_MH     ------------------------------------------------------------------------------------------------------------------------------------------------------
exu_FR     ------------------------------------------------------------------------------------------------------------------------------------------------------
exu_MR     ------------------------------------------------------------------------------------------------------------------------------------------------------

           ....|....|....|....|....|....|....|....|....|....|....|....|....|....|....|....|....|....|....|....|....|....|....|....|....|....|....|....|....|....|
               905       915       925       935       945       955       965       975       985       995      1005      1015      1025      1035      1045
exu_Gene   AGCACGAGCAAGAAAAGCGCGAATTTGCACGAAGCAATTGGAACAATTGAAAGAGCGCAGCATGAAGGATCAAGTTAACACCGGAAAAAATTACAATAATAGTGGATCGTAACTGGAAATTATACCGTACTGAGTTGAGTTGTTCTTACT
exu_FH     ------------------------------------------------------------------------------------------------------------------------------------------------------
exu_MH     ------------------------------------------------------------------------------------------------------------------------------------------------------
exu_FR     ------------------------------------------------------------------------------------------------------------------------------------------------------
exu_MR     ------------------------------------------------------------------------------------------------------------------------------------------------------

           ....|....|....|....|....|....|....|....|....|....|....|....|....|....|....|....|....|....|....|....|....|....|....|....|....|....|....|....|....|....|
              1055      1065      1075      1085      1095      1105      1115      1125      1135      1145      1155      1165      1175      1185      1195
exu_Gene   CCACTTCCGCCCCACGTCCTTACCACCGTTTGTCGAAACGATTCTTGATGCACCTACTGCGCTTACTTCCGTTTGAGATTCGACAATATATGTGCATATATACATACATACTTATGTATGTATATATCAGCAGCTATACAAAGCAAAGTG
exu_FH     ------------------------------------------------------------------------------------------------------------------------------------------------------
exu_MH     ------------------------------------------------------------------------------------------------------------------------------------------------------
exu_FR     ------------------------------------------------------------------------------------------------------------------------------------------------------
exu_MR     ------------------------------------------------------------------------------------------------------------------------------------------------------
```

```
         ....|....|....|....|....|....|....|....|....|....|....|....|....|....|....|....|....|....|....|....|....|....|....|....|....|....|....|....|....|....|
            1205      1215      1225      1235      1245      1255      1265      1275      1285      1295      1305      1315      1325      1335      1345
exu_Gene AGTGAGTAAAGTTACGACGTTGGCTTGAAATGTCTAGCGCTGACAGCGGCTAGTTTATTTGATCCGTTGAAGTCAAACTGCGTGGTTCGCGTATACACAAACAGTGTACAAACTCTCAAAGTTGTACCAAAATTACAGTTCTGTTAGTTG
exu_FH   ------------------------------------------------------------------------------------------------------------------------------------------------------
exu_MH   ------------------------------------------------------------------------------------------------------------------------------------------------------
exu_FR   -----------------------------------------------------TTTATTTGATCCGTTGAAGTCAAACTGCGTGGTTCGCGTATACACAAACAGTGTACAAACTCTCAAAGTTGTACCAAAATTACAGTTCT--------
exu_MR   ------------------------------------------------------------------------------------------------------------------------------------------------------

         ....|....|....|....|....|....|....|....|....|....|....|....|....|....|....|....|....|....|....|....|....|....|....|....|....|....|....|....|....|....|
            1355      1365      1375      1385      1395      1405      1415      1425      1435      1445      1455      1465      1475      1485      1495
exu_Gene GGATTTTTTTTTTTTATCGTTATTAAGCAAGCAATCAAACATGTGATAGACTACTCAAACTTCGGAGTTTTCTTTTCAAATTGTTTTGCATTTCTATTCAAGTTTTATTTTTTTAAATTTTAAGAAAAATCACTTGCTAAATTCAAGTTT
exu_FH   ------------------------------------------------------------------------------------------------------------------------------------------------------
exu_MH   ------------------------------------------------------------------------------------------------------------------------------------------------------
exu_FR   ------------------------------------------------------------------------------------------------------------------------------------------------------
exu_MR   -------------------------------------------------------------------TTTTCTTTTCAAATTGTTTTGCATTTCTATTCAAGTTTTATTTTTTTAAATTTTAAGAAAAATCACTTGCTAAATTCAAGTTT

         ....|....|....|....|....|....|....|....|....|....|....|....|....|....|....|....|....|....|....|....|....|....|....|....|....|....|....|....|....|....|
            1505      1515      1525      1535      1545      1555      1565      1575      1585      1595      1605      1615      1625      1635      1645
exu_Gene CTTTCTATATTCACAGGGATTAGATTGTCTTTAAGAAAAATTTCGATTCAGTACGTTCAGAAAAATTCGTTTGTTAGCAGAGAGAAAGGAGTTCAGCTGACTCAGGTAATGGCGGGAGAGGGCACAAAAATTGTCTGACCAATTGCAATG
exu_FH   ----------------GGATTAGATTGTCTTTAAGAAAAATTTCGATTCAGTACGTTCAGAAAAATTCGTTTGTTAGCAGAGAGAAAGGAGTTCAGCTGACTCAG---------------------------------------------
exu_MH   ----------------GGATTAGATTGTCTTTAAGAAAAATTTCGATTCAGTACGTTCAGAAAAATTCGTTTGTTAGCAGAGAGAAAGGAGTTCAGCTGACTCAG---------------------------------------------
exu_FR   ----------------GGATTAGATTGTCTTTAAGAAAAATTTCGATTCAGTACGTTCAGAAAAATTCGTTTGTTAGCAGAGAGAAAGGAGTTCAGCTGACTCAG---------------------------------------------
exu_MR   CTTTCTATATTCACAGGGATTAGATTGTCTTTAAGAAAAATTTCGATTCAGTACGTTCAGAAAAATTCGTTTGTTAGCAGAGAGAAAGGAGTTCAGCTGACTCAG---------------------------------------------

         ....|....|....|....|....|....|....|....|....|....|....|....|....|....|....|....|....|....|....|....|....|....|....|....|....|....|....|....|....|....|
            1655      1665      1675      1685      1695      1705      1715      1725      1735      1745      1755      1765      1775      1785      1795
exu_Gene GGCTGGCGTTAATATTTTTTTAAAATTTAAAACCAAATTTAAAGAAAAGTAATTTTGATTCAAGAAAGGAACTAAAATGCAATAATGATTTATGAGATTTTTTTGCTCGTTCATTTAATTAATGGTGAATTTTTTTTTATTTTTGCTACT
exu_FH   ------------------------------------------------------------------------------------------------------------------------------------------------------
exu_MH   ------------------------------------------------------------------------------------------------------------------------------------------------------
exu_FR   ------------------------------------------------------------------------------------------------------------------------------------------------------
exu_MR   ------------------------------------------------------------------------------------------------------------------------------------------------------
```

```
          ....|....|....|....|....|....|....|....|....|....|....|....|....|....|....|....|....|....|....|....|....|....|....|....|....|....|....|....|....|....|
             1805      1815      1825      1835      1845      1855      1865      1875      1885      1895      1905      1915      1925      1935      1945
exu_Gene  GCTTAAAGATTGCATTACAAATTGGCATATGAAATTAAAAAATTTATTTGCCTTTAAATTATTTAGTCGACAATTTTTAACACATTTTTAACCCGAAAATTCCTTTTCCTTTGTCTATTTTCAGCGCATTTAACTAGTCAAATTAGTCCG
exu_FH    ----------------------------------------------------------------------------------------------------------------------------CGCATTTAACTAGTCAAATTAGTCCG
exu_MH    ----------------------------------------------------------------------------------------------------------------------------CGCATTTAACTAGTCAAATTAGTCCG
exu_FR    ----------------------------------------------------------------------------------------------------------------------------CGCATTTAACTAGTCAAATTAGTCCG
exu_MR    ----------------------------------------------------------------------------------------------------------------------------CGCATTTAACTAGTCAAATTAGTCCG

          ....|....|....|....|....|....|....|....|....|....|....|....|....|....|....|....|....|....|....|....|....|....|....|....|....|....|....|....|....|....|
             1955      1965      1975      1985      1995      2005      2015      2025      2035      2045      2055      2065      2075      2085      2095
exu_Gene  ATTTCGCTTGGATTATTTTTATTGGAAATTTTAGAAAATGGTCGCTTCAACGAATGCAAGCCAAAACGGTGTAGTCAATTCTCCAGTAGTAGGAACTTCTAATGGAGTAACAACCAATGGCACAATGAATGTCGCTGGCGGACACAAGAC
exu_FH    ATTTCGCTTGGATTATTTTTATTGGAAATTTTAGAAAATGGTCGCTTCAACGAATGCAAGCCAAAACGGTGTAGTCAATTCTCCAGTAGTAGGAACTTCTAATGGAGTAACAACCAATGGCACAATGAATGTCGCTGGCGGACACAAGAC
exu_MH    ATTTCGCTTGGATTATTTTTATTGGAAATTTTAGAAAATGGTCGCTTCAACGAATGCAAGCCAAAACGGTGTAGTCAATTCTCCAGTAGTAGGAACTTCTAATGGAGTAACAACCAATGGCACAATGAATGTCGCTGGCGGACACAAGAC
exu_FR    ATTTCGCTTGGATTATTTTTATTGGAAATTTTAGAAAATGGTCGCTTCAACGAATGCAAGCCAAAACGGTGTAGTCAATTCTCCAGTAGTAGGAACTTCTAATGGAGTAACAACCAATGGCACAATGAATGTCGCTGGCGGACACAAGAC
exu_MR    ATTTCGCTTGGATTATTTTTATTGGAAATTTTAGAAAATGGTCGCTTCAACGAATGCAAGCCAAAACGGTGTAGTCAATTCTCCAGTAGTAGGAACTTCTAATGGAGTAACAACCAATGGCACAATGAATGTCGCTGGCGGACACAAGAC

          ....|....|....|....|....|....|....|....|....|....|....|....|....|....|....|....|....|....|....|....|....|....|....|....|....|....|....|....|....|....|
             2105      2115      2125      2135      2145      2155      2165      2175      2185      2195      2205      2215      2225      2235      2245
exu_Gene  TTTGGCCAAGGGCAGGTACACTTTAGTAGGCGTCGATATTGACACAACTGGGCGTCGACTCATCGATGAAGTAAGTATGCGTGTGTGTGTTATAAATGTTTATTATACATATAATTTAAACAATCAGAACACAGTTTGTCCAATTCCT
exu_FH    TTTGGCCAAGGGCAGGTACACTTTAGTAGGCGTCGATATTGACACAACTGGGCGTCGACTCATCGATGAA--------------------------------------------------------------------------------
exu_MH    TTTGGCCAAGGGCAGGTACACTTTAGTAGGCGTCGATATTGACACAACTGGGCGTCGACTCATCGATGAA--------------------------------------------------------------------------------
exu_FR    TTTGGCCAAGGGCAGGTACACTTTAGTAGGCGTCGATATTGACACAACTGGGCGTCGACTCATCGATGAA--------------------------------------------------------------------------------
exu_MR    TTTGGCCAAGGGCAGGTACACTTTAGTAGGCGTCGATATTGACACAACTGGGCGTCGACTCATCGATGAA--------------------------------------------------------------------------------

          ....|....|....|....|....|....|....|....|....|....|....|....|....|....|....|....|....|....|....|....|....|....|....|....|....|....|....|....|....|....|
             2255      2265      2275      2285      2295      2305      2315      2325      2335      2345      2355      2365      2375      2385      2395
exu_Gene  ATCCTTATAATAACATTTGCATTTCTAGAACTTCTCACCTAAAAATTTGTTTAAGCCGACTAGCGGCTCTTTTAACTTTTAATTTAATTTTAATATAAATTTGTAATTATTTTCTTGTATATACAAACGATTAATTTTAGTTTGTTATAA
exu_FH    ------------------------------------------------------------------------------------------------------------------------------------------------------
exu_MH    ------------------------------------------------------------------------------------------------------------------------------------------------------
exu_FR    ------------------------------------------------------------------------------------------------------------------------------------------------------
exu_MR    ------------------------------------------------------------------------------------------------------------------------------------------------------
```

```
          ....|....|....|....|....|....|....|....|....|....|....|....|....|....|....|....|....|....|....|....|....|....|....|....|....|....|....|....|....|....|
              2405      2415      2425      2435      2445      2455      2465      2475      2485      2495      2505      2515      2525      2535      2545
exu_Gene  GGGAAGCAAAATATGTGAAAATCCCCGGCTTCAGTTTGCAAGAATGTGCATAAAAACTGTTCATATGCGCATGGAAAATATATTTTTTTAAAAAAAAACTATATTAATGCATCCGCTTTGCAGGTAATCCGCTTTACTGTTACAAAGTCA
exu_FH    ------------------------------------------------------------------------------------------------------------------------------------------------------
exu_MH    ------------------------------------------------------------------------------------------------------------------------------------------------------
exu_FR    ------------------------------------------------------------------------------------------------------------------------------------------------------
exu_MR    ------------------------------------------------------------------------------------------------------------------------------------------------------

          ....|....|....|....|....|....|....|....|....|....|....|....|....|....|....|....|....|....|....|....|....|....|....|....|....|....|....|....|....|....|
              2555      2565      2575      2585      2595      2605      2615      2625      2635      2645      2655      2665      2675      2685      2695
exu_Gene  TGAACTTGTGCGTATGGGTGATGCATTACTTGAATATGTTGTTTGTGCATTGATAACTGCACTTTTTTTTCCTGATTCGGCTTTTGGTTTTCGTCTAATTTTACTATTCCCCTACAAGTCATTGGCCTTTGAAATCGTTTACTTTCTTTG
exu_FH    ------------------------------------------------------------------------------------------------------------------------------------------------------
exu_MH    ------------------------------------------------------------------------------------------------------------------------------------------------------
exu_FR    ------------------------------------------------------------------------------------------------------------------------------------------------------
exu_MR    ------------------------------------------------------------------------------------------------------------------------------------------------------

          ....|....|....|....|....|....|....|....|....|....|....|....|....|....|....|....|....|....|....|....|....|....|....|....|....|....|....|....|....|....|
              2705      2715      2725      2735      2745      2755      2765      2775      2785      2795      2805      2815      2825      2835      2845
exu_Gene  CGCAAATTATACTTTTTTTGTTGTTGTTTTATTTATTTATTTTAACCTTATGGCCGATGTACATATATACAAATATGTAAATATGTAATATATATTCCAAAACTAAAACTTCCTCAATTGCTTTGTCAAAGTTTGTTTTCCGGGTTTTTT
exu_FH    ------------------------------------------------------------------------------------------------------------------------------------------------------
exu_MH    ------------------------------------------------------------------------------------------------------------------------------------------------------
exu_FR    ------------------------------------------------------------------------------------------------------------------------------------------------------
exu_MR    ------------------------------------------------------------------------------------------------------------------------------------------------------

          ....|....|....|....|....|....|....|....|....|....|....|....|....|....|....|....|....|....|....|....|....|....|....|....|....|....|....|....|....|....|
              2855      2865      2875      2885      2895      2905      2915      2925      2935      2945      2955      2965      2975      2985      2995
exu_Gene  TTTTTTTTTTTTTTTATTTTTAACAAATTTTTCTTCTCAGTTATAAGAGGTTAGGAAAGCATAAAATTGTGATGCCTTATTTTGCTCTCTTCTTTTCTCCTCCACAGATCGTTCAGTTGGCCGCGTATTCGCCCAAAGATCATTTTGAAC
exu_FH    -----------------------------------------------------------------------------------------------------------ATCGTTCAGTTGGCCGCGTATTCGCCCAAAGATCATTTTGAAC
exu_MH    -----------------------------------------------------------------------------------------------------------ATCGTTCAGTTGGCCGCGTATTCGCCCAAAGATCATTTTGAAC
exu_FR    -----------------------------------------------------------------------------------------------------------ATCGTTCAGTTGGCCGCGTATTCGCCCAAAGATCATTTTGAAC
exu_MR    -----------------------------------------------------------------------------------------------------------ATCGTTCAGTTGGCCGCGTATTCGCCCAAAGATCATTTTGAAC
```

```
          ....|....|....|....|....|....|....|....|....|....|....|....|....|....|....|....|....|....|....|....|....|....|....|....|....|....|....|....|....|....|
             3005      3015      3025      3035      3045      3055      3065      3075      3085      3095      3105      3115      3125      3135      3145
exu_Gene  AATATATTATGCCATACATGAACTTGAATCCTGCTGCTCGCCAACGTCACCAAGTGCGTGTGATCTCTATTGGATTCTATCGTATGTTGAAATCAATGCAAACCTATAAGGTGAGAGAAAAAAAAATACATTAAGTGACTGGACTTCATT
exu_FH    AATATATTATGCCATACATGAACTTGAATCCTGCTGCTCGCCAACGTCACCAAGTGCGTGTGATCTCTATTGGATTCTATCGTATGTTGAAATCAATGCAAACCTATAAG----------------------------------------
exu_MH    AATATATTATGCCATACATGAACTTGAATCCTGCTGCTCGCCAACGTCACCAAGTGCGTGTGATCTCTATTGGATTCTATCGTATGTTGAAATCAATGCAAACCTATAAG----------------------------------------
exu_FR    AATATATTATGCCATACATGAACTTGAATCCTGCTGCTCGCCAACGTCACCAAGTGCGTGTGATCTCTATTGGATTCTATCGTATGTTGAAATCAATGCAAACCTATAAG----------------------------------------
exu_MR    AATATATTATGCCATACATGAACTTGAATCCTGCTGCTCGCCAACGTCACCAAGTGCGTGTGATCTCTATTGGATTCTATCGTATGTTGAAATCAATGCAAACCTATAAG----------------------------------------

          ....|....|....|....|....|....|....|....|....|....|....|....|....|....|....|....|....|....|....|....|....|....|....|....|....|....|....|....|....|....|
             3155      3165      3175      3185      3195      3205      3215      3225      3235      3245      3255      3265      3275      3285      3295
exu_Gene  TATGCACCTCCAATTACGTTTGATTGGTAGTTGCCATATTTTTTTTTTTTGTGTAATTAACAAAAAATATATATTGTTGACGTTGTTGCATACAAAAGTGTAAATACAATTACTAAAAATATTTTTTATATAAAAGGTTTTCCAATAAAA
exu_FH    ------------------------------------------------------------------------------------------------------------------------------------------------------
exu_MH    ------------------------------------------------------------------------------------------------------------------------------------------------------
exu_FR    ------------------------------------------------------------------------------------------------------------------------------------------------------
exu_MR    ------------------------------------------------------------------------------------------------------------------------------------------------------

          ....|....|....|....|....|....|....|....|....|....|....|....|....|....|....|....|....|....|....|....|....|....|....|....|....|....|....|....|....|....|
             3305      3315      3325      3335      3345      3355      3365      3375      3385      3395      3405      3415      3425      3435      3445
exu_Gene  GCTGTTATTTTGATATTCCAAAAAAATTTTTTTTTTTTATTAAAAAATGATCGGATGTTTATTTCATTATAAAGCGAAAGGTATGCCGGGACTAGGGAAAAATAACGTCGGGCAAATGACCACCACAACCACGCGTTTCCACGGCAGTCG
exu_FH    ------------------------------------------------------------------------------------------------------------------------------------------------------
exu_MH    ------------------------------------------------------------------------------------------------------------------------------------------------------
exu_FR    ------------------------------------------------------------------------------------------------------------------------------------------------------
exu_MR    ------------------------------------------------------------------------------------------------------------------------------------------------------

          ....|....|....|....|....|....|....|....|....|....|....|....|....|....|....|....|....|....|....|....|....|....|....|....|....|....|....|....|....|....|
             3455      3465      3475      3485      3495      3505      3515      3525      3535      3545      3555      3565      3575      3585      3595
exu_Gene  GTTCTACGTTACCAAAACAACCCGGATTTATATCCGGCCAAGGATTGCCACTCCAGCAGCTTTCCCCGTATGTAAAAAGGGGGAATGTTTATGCTGATACAACAACAACAACAACCACGCTTGCCACGCGTTTCCACGGCAGTCGGTTCT
exu_FH    ------------------------------------------------------------------------------------------------------------------------------------------------------
exu_MH    ------------------------------------------------------------------------------------------------------------------------------------------------------
exu_FR    ------------------------------------------------------------------------------------------------------------------------------------------------------
exu_MR    ------------------------------------------------------------------------------------------------------------------------------------------------------
```

```
           ....|....|....|....|....|....|....|....|....|....|....|....|....|....|....|....|....|....|....|....|....|....|....|....|....|....|....|....|....|....|
               3605      3615      3625      3635      3645      3655      3665      3675      3685      3695      3705      3715      3725      3735      3745
exu_Gene   ACGTTACTGAAACAATCCGGATTTATATCCGGCCAAGGATTTCCACTCCAGCAGCTTTCCCCGTATGTAAAAATGGAAATTGTTTATGCTGATACAACAACAACAACAAAAACCACGCTTCCAGGACAATATCCTTTTCATGAATTGTTC
exu_FH     ------------------------------------------------------------------------------------------------------------------------------------------------------
exu_MH     ------------------------------------------------------------------------------------------------------------------------------------------------------
exu_FR     ------------------------------------------------------------------------------------------------------------------------------------------------------
exu_MR     ------------------------------------------------------------------------------------------------------------------------------------------------------

           ....|....|....|....|....|....|....|....|....|....|....|....|....|....|....|....|....|....|....|....|....|....|....|....|....|....|....|....|....|....|
               3755      3765      3775      3785      3795      3805      3815      3825      3835      3845      3855      3865      3875      3885      3895
exu_Gene   CATAACCAAATTGCAAAGTGGCTGCCCTATATCCTCGATAGCCTCACGAATTCCATCTTTGAGGTCTTGAATCCTCCCTGGGCTGTTGGCGTAAACCTTCTCATTCACGTGGCCCCAAAAAAAAAGTTACAAGGTGTAAATTACAAAACT
exu_FH     ------------------------------------------------------------------------------------------------------------------------------------------------------
exu_MH     ------------------------------------------------------------------------------------------------------------------------------------------------------
exu_FR     ------------------------------------------------------------------------------------------------------------------------------------------------------
exu_MR     ------------------------------------------------------------------------------------------------------------------------------------------------------

           ....|....|....|....|....|....|....|....|....|....|....|....|....|....|....|....|....|....|....|....|....|....|....|....|....|....|....|....|....|....|
               3905      3915      3925      3935      3945      3955      3965      3975      3985      3995      4005      4015      4025      4035      4045
exu_Gene   CGGGGGCAATTGTGATCCCTCTTCNNNNNNNNNNNNNNNNNNNNNNNNNNNNNNNNNNNNNNNNNNNNNNNNNNNNNNNNNNNNNNNNNNNNNNNNNNNNNNNNNNNNNNNNNNNNNNNNNNNNNNNNNNNNNNNNNNNNNNNNNN
exu_FH     ------------------------------------------------------------------------------------------------------------------------------------------------------
exu_MH     ------------------------------------------------------------------------------------------------------------------------------------------------------
exu_FR     ------------------------------------------------------------------------------------------------------------------------------------------------------
exu_MR     ------------------------------------------------------------------------------------------------------------------------------------------------------

           ....|....|....|....|....|....|....|....|....|....|....|....|....|....|....|....|....|....|....|....|....|....|....|....|....|....|....|....|....|....|
               4055      4065      4075      4085      4095      4105      4115      4125      4135      4145      4155      4165      4175      4185      4195
exu_Gene   NNNNNNNNNNNNNNNNNNNNNNNNNNNNNNNNNNNNNNNNNNNNNNNNNNNNNNNNNNNNNNNNNNNNNNNNNNNNNNNNNNNNNNNNNNNNNNNNNNNNNNNNNNNNNNNNNNNNNNNNNNNNNNNNNNNNNNNNNNNNNNNNNN
exu_FH     ------------------------------------------------------------------------------------------------------------------------------------------------------
exu_MH     ------------------------------------------------------------------------------------------------------------------------------------------------------
exu_FR     ------------------------------------------------------------------------------------------------------------------------------------------------------
exu_MR     ------------------------------------------------------------------------------------------------------------------------------------------------------
```

```
                   ....|....|....|....|....|....|....|....|....|....|....|....|....|....|....|....|....|....|....|....|....|....|....|....|....|....|....|....|....|....|
                      4205      4215      4225      4235      4245      4255      4265      4275      4285      4295      4305      4315      4325      4335      4345
exu_Gene           NNNNNNNNNNNNNNNNNNNNNNNNNNNNNNNNNNNNNNNNNNNNNNNNNNNNNNNNNNNNNNNNNNNNNNNNNNNNNNNNNNNNNNNNNNNNNNNNNNNNNNNNNNNNNNNNNNNNNNNNNNNNNNNNNNNNNNNNNNNNNNNNNNNNNN
exu_FH             ------------------------------------------------------------------------------------------------------------------------------------------------------
exu_MH             ------------------------------------------------------------------------------------------------------------------------------------------------------
exu_FR             ------------------------------------------------------------------------------------------------------------------------------------------------------
exu_MR             ------------------------------------------------------------------------------------------------------------------------------------------------------

                   ....|....|....|....|....|....|....|....|....|....|....|....|....|....|....|....|....|....|....|....|....|....|....|....|....|....|....|....|....|....|
                      4355      4365      4375      4385      4395      4405      4415      4425      4435      4445      4455      4465      4475      4485      4495
exu_Gene           NNNNNNNNNNNNNNNNNNNNNNNNNNNNNNNNNNNNNNNNNNNNNNNNNNNNNNNNNNNNNNNNNNNNNNNNNNNNNNNNNNNNNNNNNNNNNNNNNNNNNNNNNNNNNNNNNNNNNNNNNNNNNNNNNNNNNNNNNNNNNNNNNNNNNN
exu_FH             ------------------------------------------------------------------------------------------------------------------------------------------------------
exu_MH             ------------------------------------------------------------------------------------------------------------------------------------------------------
exu_FR             ------------------------------------------------------------------------------------------------------------------------------------------------------
exu_MR             ------------------------------------------------------------------------------------------------------------------------------------------------------

                   ....|....|....|....|....|....|....|....|....|....|....|....|....|....|....|....|....|....|....|....|....|....|....|....|....|....|....|....|....|....|
                      4505      4515      4525      4535      4545      4555      4565      4575      4585      4595      4605      4615      4625      4635      4645
exu_Gene           NNNNNNNNNNNNNNNNNNNNNNNNNNNNNNNNNNNNNNNNNNNNNNNNNNNNNNNNNNNNNNNNNNNNNNNNNNNNNNNNNNNNNNNNNNNNNNNNNNNNNNNNNNNNNNNNNNNNNNNNNNNNNNNNNNNNNNNNNNNNNNNNNNNNNNN
exu_FH             ------------------------------------------------------------------------------------------------------------------------------------------------------
exu_MH             ------------------------------------------------------------------------------------------------------------------------------------------------------
exu_FR             ------------------------------------------------------------------------------------------------------------------------------------------------------
exu_MR             ------------------------------------------------------------------------------------------------------------------------------------------------------

                   ....|....|....|....|....|....|....|....|....|....|....|....|....|....|....|....|....|....|....|....|....|....|....|....|....|....|....|....|....|....|
                      4655      4665      4675      4685      4695      4705      4715      4725      4735      4745      4755      4765      4775      4785      4795
exu_Gene           NNNNNNNNNNNNNNNNNNNNNNNNNNNNNNNNNNNNNNNNNNNNNNNNNNNNNNNNNNNNNNNNNNNNNNNNNNNNNNNNNNNNNNNNNNNNNNNNNNNNNNNNNNNNNNNNNNNNNNNNNNNNNNNNNNNNNNNNNNNNNNNNNNNNNN
exu_FH             ------------------------------------------------------------------------------------------------------------------------------------------------------
exu_MH             ------------------------------------------------------------------------------------------------------------------------------------------------------
exu_FR             ------------------------------------------------------------------------------------------------------------------------------------------------------
exu_MR             ------------------------------------------------------------------------------------------------------------------------------------------------------
```

```
          ....|....|....|....|....|....|....|....|....|....|....|....|....|....|....|....|....|....|....|....|....|....|....|....|....|....|....|....|....|....|
              4805      4815      4825      4835      4845      4855      4865      4875      4885      4895      4905      4915      4925      4935      4945
exu_Gene  NNNNNNNNNNNNNNNNNNNNNNNNNNNNNNNNNNNNNNNNNNNNNNNNNNNNNNNNNNNNNNNNNNNNNNNNNNNNNNNNNNNNNNNNNNNNNNNNNNNNNNNNNNNNNNNNNNNNNNNNNNNNNNNNNNNNNNNNNNNNNNNNNNNNNNN
exu_FH    ------------------------------------------------------------------------------------------------------------------------------------------------------
exu_MH    ------------------------------------------------------------------------------------------------------------------------------------------------------
exu_FR    ------------------------------------------------------------------------------------------------------------------------------------------------------
exu_MR    ------------------------------------------------------------------------------------------------------------------------------------------------------

          ....|....|....|....|....|....|....|....|....|....|....|....|....|....|....|....|....|....|....|....|....|....|....|....|....|....|....|....|....|....|
              4955      4965      4975      4985      4995      5005      5015      5025      5035      5045      5055      5065      5075      5085      5095
exu_Gene  NNNNNNNNNNNNNNNNNNNNNNNNNNNNNNNNNNNNNNNNNNNNNNNNNNNNNNNNNNNNNNNNNNNNNNNNNNNNNNNNNNNNNNNNNNNNNNNNNNNNNNNNNNNNNNNNNNNNNNNNNNNNNNNNNNNNNNNNNNNNNNNNNNNNNNN
exu_FH    ------------------------------------------------------------------------------------------------------------------------------------------------------
exu_MH    ------------------------------------------------------------------------------------------------------------------------------------------------------
exu_FR    ------------------------------------------------------------------------------------------------------------------------------------------------------
exu_MR    ------------------------------------------------------------------------------------------------------------------------------------------------------

          ....|....|....|....|....|....|....|....|....|....|....|....|....|....|....|....|....|....|....|....|....|....|....|....|....|....|....|....|....|....|
              5105      5115      5125      5135      5145      5155      5165      5175      5185      5195      5205      5215      5225      5235      5245
exu_Gene  NNNNNNNNNNNNNNNNNNNNNNNNNNNNNNNNNNNNNNNNNNNNNNNNNNNNNNNNNNNNNNNNNNNNNNNNNNNNNNNNNNNNNNNNNNNNNNNNNNNNNNNNNNNNNNNNNNNNNNNNNNNNNNNNNNNNNNNNNNNNNNNNNNNNNNN
exu_FH    ------------------------------------------------------------------------------------------------------------------------------------------------------
exu_MH    ------------------------------------------------------------------------------------------------------------------------------------------------------
exu_FR    ------------------------------------------------------------------------------------------------------------------------------------------------------
exu_MR    ------------------------------------------------------------------------------------------------------------------------------------------------------

          ....|....|....|....|....|....|....|....|....|....|....|....|....|....|....|....|....|....|....|....|....|....|....|....|....|....|....|....|....|....|
              5255      5265      5275      5285      5295      5305      5315      5325      5335      5345      5355      5365      5375      5385      5395
exu_Gene  NNNNNNNNNNNNNNNNNNNNNNNNNNNNNNNNNNNNNNNNNNNNNNNNNNNNNNNNNNNNNNNNNNNNNNNNNNNNNNNNNNNNNNNNNNNNNNNNNNNNNNNNNNNNNNNNNNNNNNNNNNNNNNNNNNNNNNNNNNNNNNNNNNNNNN
exu_FH    ------------------------------------------------------------------------------------------------------------------------------------------------------
exu_MH    ------------------------------------------------------------------------------------------------------------------------------------------------------
exu_FR    ------------------------------------------------------------------------------------------------------------------------------------------------------
exu_MR    ------------------------------------------------------------------------------------------------------------------------------------------------------
```

```
          ....|....|....|....|....|....|....|....|....|....|....|....|....|....|....|....|....|....|....|....|....|....|....|....|....|....|....|....|....|....|
              5405      5415      5425      5435      5445      5455      5465      5475      5485      5495      5505      5515      5525      5535      5545
exu_Gene  NNNNNNNNNNNNNNNNNNNNNNNNNNNNNNNNNNNNNNNNNNNNNNNNNNNNNNNNNNNNNNNNNNNNNNNNNNNNNNNNNNNNNNNNNNNNNNNNNNNNNNNNNNNNNNNNNNNNNNNNNNNNNNNNNNNNNNNNNNNNNNNNNNNNNN
exu_FH    ------------------------------------------------------------------------------------------------------------------------------------------------------
exu_MH    ------------------------------------------------------------------------------------------------------------------------------------------------------
exu_FR    ------------------------------------------------------------------------------------------------------------------------------------------------------
exu_MR    ------------------------------------------------------------------------------------------------------------------------------------------------------

          ....|....|....|....|....|....|....|....|....|....|....|....|....|....|....|....|....|....|....|....|....|....|....|....|....|....|....|....|....|....|
              5555      5565      5575      5585      5595      5605      5615      5625      5635      5645      5655      5665      5675      5685      5695
exu_Gene  NNNNNNNNNNNNNNNNNNNNNNNNNNNNNNNNNNNNNNNNNNNNNNNNNNNNNNNNNNNNNNNNNNNNNNNNNNNNNNNNNNNNNNNNNNNNNNNNNNNNNNNNNNNNNNNNNNNNNNNNNNNNNNNNTTGTTTCTTGAAACTTTAAACG
exu_FH    ------------------------------------------------------------------------------------------------------------------------------------------------------
exu_MH    ------------------------------------------------------------------------------------------------------------------------------------------------------
exu_FR    ------------------------------------------------------------------------------------------------------------------------------------------------------
exu_MR    ------------------------------------------------------------------------------------------------------------------------------------------------------

          ....|....|....|....|....|....|....|....|....|....|....|....|....|....|....|....|....|....|....|....|....|....|....|....|....|....|....|....|....|....|
              5705      5715      5725      5735      5745      5755      5765      5775      5785      5795      5805      5815      5825      5835      5845
exu_Gene  CGTTTTTTTTGGAACTGGGTTTTCAAAGTCGGTTGTCAAAAGTTTCTGAAAACTATTCAACCGATCTTGATGAAATTTTCACCGGTGTTAGAGATACAATTTATTCGTGCTTGAACGAAGGGTTTTTTTTTTTTTTTTTCAATTACAACA
exu_FH    ------------------------------------------------------------------------------------------------------------------------------------------------------
exu_MH    ------------------------------------------------------------------------------------------------------------------------------------------------------
exu_FR    ------------------------------------------------------------------------------------------------------------------------------------------------------
exu_MR    ------------------------------------------------------------------------------------------------------------------------------------------------------

          ....|....|....|....|....|....|....|....|....|....|....|....|....|....|....|....|....|....|....|....|....|....|....|....|....|....|....|....|....|....|
              5855      5865      5875      5885      5895      5905      5915      5925      5935      5945      5955      5965      5975      5985      5995
exu_Gene  ATTTAAAGAAAAACAAAATGTCGAGCAAATTTGACCGAAATTTTCATTTTTTTGCATAAATGTCTGCCAAAATTCCAATTTTTACTTTTTTTTCTTTCCTTCGTTCAAGCTCGAGTTTATCGTCTTAACTAAAGCACTAATTTTTGTTTT
exu_FH    ------------------------------------------------------------------------------------------------------------------------------------------------------
exu_MH    ------------------------------------------------------------------------------------------------------------------------------------------------------
exu_FR    ------------------------------------------------------------------------------------------------------------------------------------------------------
exu_MR    ------------------------------------------------------------------------------------------------------------------------------------------------------
```

```
         ....|....|....|....|....|....|....|....|....|....|....|....|....|....|....|....|....|....|....|....|....|....|....|....|....|....|....|....|....|....|
             6005      6015      6025      6035      6045      6055      6065      6075      6085      6095      6105      6115      6125      6135      6145
exu_Gene TTCATTTTTGATGAGCCTGTCAGGAGTTATGCTGACAACGCGGACGCATCTTTTTTTCGAGGGGTCACCGGCTTGACGTCACAATGGAGGAGCTTTAATTTTTTGAAATTATTCTTTAAACATGTATCTATAAGACAGAAAAAAGATTGA
exu_FH   ------------------------------------------------------------------------------------------------------------------------------------------------------
exu_MH   ------------------------------------------------------------------------------------------------------------------------------------------------------
exu_FR   ------------------------------------------------------------------------------------------------------------------------------------------------------
exu_MR   ------------------------------------------------------------------------------------------------------------------------------------------------------

         ....|....|....|....|....|....|....|....|....|....|....|....|....|....|....|....|....|....|....|....|....|....|....|....|....|....|....|....|....|....|
             6155      6165      6175      6185      6195      6205      6215      6225      6235      6245      6255      6265      6275      6285      6295
exu_Gene ATTAAATAAATAATTTTTTACATAGAAAAAAAATATATTGAAAATAGGCCTTTTTTTACTCGACAAAACCCATGTAACTCCTTAATGCCAACCAGACTAAACTAACCTTATCTGGAAGCTTGAGACGTTAGCTGTAATAGAAATGTGTTA
exu_FH   ------------------------------------------------------------------------------------------------------------------------------------------------------
exu_MH   ------------------------------------------------------------------------------------------------------------------------------------------------------
exu_FR   ------------------------------------------------------------------------------------------------------------------------------------------------------
exu_MR   ------------------------------------------------------------------------------------------------------------------------------------------------------

         ....|....|....|....|....|....|....|....|....|....|....|....|....|....|....|....|....|....|....|....|....|....|....|....|....|....|....|....|....|....|
             6305      6315      6325      6335      6345      6355      6365      6375      6385      6395      6405      6415      6425      6435      6445
exu_Gene TATTCACGTCCAAAGTCGAAAAAGCCAAACCGGGTGCCCAACGGCGGATTACATTTTTTTTTTCGTTTTTCTTATAACTTTGAATTTCGCATTTATTTAATTTCTTTATTTTTTAACAGTGTTCTCTGATTTAAAATTTGTAATACTTAA
exu_FH   ------------------------------------------------------------------------------------------------------------------------------------------------------
exu_MH   ------------------------------------------------------------------------------------------------------------------------------------------------------
exu_FR   ------------------------------------------------------------------------------------------------------------------------------------------------------
exu_MR   ------------------------------------------------------------------------------------------------------------------------------------------------------

         ....|....|....|....|....|....|....|....|....|....|....|....|....|....|....|....|....|....|....|....|....|....|....|....|....|....|....|....|....|....|
             6455      6465      6475      6485      6495      6505      6515      6525      6535      6545      6555      6565      6575      6585      6595
exu_Gene AATTAAATGAATATAACGAATTGTCTAGTCTAGTTTTAAGTATTACGCATAAAATGCCGTAATTATTAATAAATAAAATAAAATGCATTTATATTCCTCAGATTATTAAGTCGCAATTATGAATAAATAACATAAAATAAAGTGCATTTC
exu_FH   ------------------------------------------------------------------------------------------------------------------------------------------------------
exu_MH   ------------------------------------------------------------------------------------------------------------------------------------------------------
exu_FR   ------------------------------------------------------------------------------------------------------------------------------------------------------
exu_MR   ------------------------------------------------------------------------------------------------------------------------------------------------------
```

```
         ....|....|....|....|....|....|....|....|....|....|....|....|....|....|....|....|....|....|....|....|....|....|....|....|....|....|....|....|....|....|
              6605      6615      6625      6635      6645      6655      6665      6675      6685      6695      6705      6715      6725      6735      6745
exu_Gene TATTCCTCAGATTATCAAGTCTAAATCGGAGATCGCTGCCTTGAAAGATTTTCTGGATTGGCTGGAAGCGCTTAAGGCCGAAGATACATCATCGAACGGTGTCATTCTAGTCTATCATGAGGAACGCAAATTTATTCCATATATGATTTT
exu_FH   ----------ATTATCAAGTCTAAATCGGAGATCGCTGCCTTGAAAGATTTTCTGGATTGGCTGGAAGCGCTTAAGGCCGAAGATACATCATCGAACGGTGTCATTCTAGTCTATCATGAGGAACGCAAATTTATTCCATATATGATTTT
exu_MH   ----------ATTATCAAGTCTAAATCGGAGATCGCTGCCTTGAAAGATTTTCTGGATTGGCTGGAAGCGCTTAAGGCCGAAGATACATCATCGAACGGTGTCATTCTAGTCTATCATGAGGAACGCAAATTTATTCCATATATGATTTT
exu_FR   ----------ATTATCAAGTCTAAATCGGAGATCGCTGCCTTGAAAGATTTTCTGGATTGGCTGGAAGCGCTTAAGGCCGAAGATACATCATCGAACGGTGTCATTCTAGTCTATCATGAGGAACGCAAATTTATTCCATATATGATTTT
exu_MR   ----------ATTATCAAGTCTAAATCGGAGATCGCTGCCTTGAAAGATTTTCTGGATTGGCTGGAAGCGCTTAAGGCCGAAGATACATCATCGAACGGTGTCATTCTAGTCTATCATGAGGAACGCAAATTTATTCCATATATGATTTT

         ....|....|....|....|....|....|....|....|....|....|....|....|....|....|....|....|....|....|....|....|....|....|....|....|....|....|....|....|....|....|
              6755      6765      6775      6785      6795      6805      6815      6825      6835      6845      6855      6865      6875      6885      6895
exu_Gene GGAATCGCTTAACAAATACAACCTGTTGGACAGGTTCCTTAAAACAGTCAAATCATTTGCCAATGGCCTCAATTTAGCCAAAGCCAATGCCAACGAATCATTGCAACATTACACGTTGCGCAAACTCTCCAAAGTGTTGAGCGCCAAAGC
exu_FH   GGAATCGCTTAACAAATACAACCTGTTGGACAGGTTCCTTAAAACAGTCAAATCATTTGCCAATGGCCTCAATTTAGCCAAAGCCAATGCCAACGAATCATTGCAACATTACACGTTGCGCAAACTCTCCAAAGTGTTGAGCGCCAAAGC
exu_MH   GGAATCGCTTAACAAATACAACCTGTTGGACAGGTTCCTTAAAACAGTCAAATCATTTGCCAATGGCCTCAATTTAGCCAAAGCCAATGCCAACGAATCATTGCAACATTACACGTTGCGCAAACTCTCCAAAGTGTTGAGCGCCAAAGC
exu_FR   GGAATCGCTTAACAAATACAACCTGTTGGACAGGTTCCTTAAAACAGTCAAATCATTTGCCAATGGCCTCAATTTAGCCAAAGCCAATGCCAACGAATCATTGCAACATTACACGTTGCGCAAACTCTCCAAAGTGTTGAGCGCCAAAGC
exu_MR   GGAATCGCTTAACAAATACAACCTGTTGGACAGGTTCCTTAAAACAGTCAAATCATTTGCCAATGGCCTCAATTTAGCCAAAGCCAATGCCAACGAATCATTGCAACATTACACGTTGCGCAAACTCTCCAAAGTGTTGAGCGCCAAAGC

         ....|....|....|....|....|....|....|....|....|....|....|....|....|....|....|....|....|....|....|....|....|....|....|....|....|....|....|....|....|....|
              6905      6915      6925      6935      6945      6955      6965      6975      6985      6995      7005      7015      7025      7035      7045
exu_Gene ACACGAAGTGATCAATAATCAAAATGCAAGTGACGGTGAAATCAAAAACAATGTTAGCTCAACAACGGGCATAGCCGCCGAGTGTCAGAGCCCCCAAAAGTCATTGCAAAAGGGCGTTCTGGCACAGGAGCGTGAGCTGTTCGATGGTAA
exu_FH   ACACGAAGTGATCAATAATCAAAATGCAAGTGACGGTGAAATCAAAAACAATGTTAGCTCAACAACGGGCATAGCCGCCGAGTGTCAGAGCCCCCAAAAGTCATTGCAAAAGGGCGTTCTGGCACAGGAGCGTGAGCTGTTCGATGGTAA
exu_MH   ACACGAAGTGATCAATAATCAAAATGCAAGTGACGGTGAAATCAAAAACAATGTTAGCTCAACAACGGGCATAGCCGCCGAGTGTCAGAGCCCCCAAAAGTCATTGCAAAAGGGCGTTCTGGCACAGGAGCGTGAGCTGTTCGATGGTAA
exu_FR   ACACGAAGTGATCAATAATCAAAATGCAAGTGACGGTGAAATCAAAAACAATGTTAGCTCAACAACGGGCATAGCCGCCGAGTGTCAGAGCCCCCAAAAGTCATTGCAAAAGGGCGTTCTGGCACAGGAGCGTGAGCTGTTCGATGGTAA
exu_MR   ACACGAAGTGATCAATAATCAAAATGCAAGTGACGGTGAAATCAAAAACAATGTTAGCTCAACAACGGGCATAGCCGCCGAGTGTCAGAGCCCCCAAAAGTCATTGCAAAAGGGCGTTCTGGCACAGGAGCGTGAGCTGTTCGATGGTAA

         ....|....|....|....|....|....|....|....|....|....|....|....|....|....|....|....|....|....|....|....|....|....|....|....|....|....|....|....|....|....|
              7055      7065      7075      7085      7095      7105      7115      7125      7135      7145      7155      7165      7175      7185      7195
exu_Gene TGCTAGTGTGCGTGCTAAGTTAGCCTACGAGGTGGCACAACAGTTGAGCAATCAAGAACACAAGGTGCCTATCGAATCGTCGGCTGCATTAGAGAATATGATTGCCGCAGTGCAAACGTATGCACAGCCCATCGAATGCGATTTGAATGA
exu_FH   TGCTAGTGTGCGTGCTAAGTTAGCCTACGAGGTGGCACAACAGTTGAGCAATCAAGAACACAAGGTGCCTATCGAATCGTCGGCTGCATTAGAGAATATGATTGCCGCAGTGCAAACGTATGCACAGCCCATCGAATGCGATTTGAATGA
exu_MH   TGCTAGTGTGCGTGCTAAGTTAGCCTACGAGGTGGCACAACAGTTGAGCAATCAAGAACACAAGGTGCCTATCGAATCGTCGGCTGCATTAGAGAATATGATTGCCGCAGTGCAAACGTATGCACAGCCCATCGAATGCGATTTGAATGA
exu_FR   TGCTAGTGTGCGTGCTAAGTTAGCCTACGAGGTGGCACAACAGTTGAGCAATCAAGAACACAAGGTGCCTATCGAATCGTCGGCTGCATTAGAGAATATGATTGCCGCAGTGCAAACGTATGCACAGCCCATCGAATGCGATTTGAATGA
exu_MR   TGCTAGTGTGCGTGCTAAGTTAGCCTACGAGGTGGCACAACAGTTGAGCAATCAAGAACACAAGGTGCCTATCGAATCGTCGGCTGCATTAGAGAATATGATTGCCGCAGTGCAAACGTATGCACAGCCCATCGAATGCGATTTGAATGA
```

```
         ....|....|....|....|....|....|....|....|....|....|....|....|....|....|....|....|....|....|....|....|....|....|....|....|....|....|....|....|....|....|
             7205      7215      7225      7235      7245      7255      7265      7275      7285      7295      7305      7315      7325      7335      7345
exu_Gene ACTACAAACTCAAAACGAGAATTTGGAGCGTCAAAACTCTTTCCGTCCGGTTTTCTTAAATTACTTTAAAACCACACTCTACCATCGAGTGCGTGCAGTCAAGTTTCGCATCATACTTGCGGAACAGGGTTATGACCTGCAGACGCTAAA
exu_FH   ACTACAAACTCAAAACGAGAATTTGGAGCGTCAAAACTCTTTCCGTCCGGTTTTCTTAAATTACTTTAAAACCACACTCTACCATCGAGTGCGTGCAGTCAAGTTTCGCATCATACTTGCGGAACAGGGTTATGACCTGCAGACGCTAAA
exu_MH   ACTACAAACTCAAAACGAGAATTTGGAGCGTCAAAACTCTTTCCGTCCGGTTTTCTTAAATTACTTTAAAACCACACTCTACCATCGAGTGCGTGCAGTCAAGTTTCGCATCATACTTGCGGAACAGGGTTATGACCTGCAGACGCTAAA
exu_FR   ACTACAAACTCAAAACGAGAATTTGGAGCGTCAAAACTCTTTCCGTCCGGTTTTCTTAAATTACTTTAAAACCACACTCTACCATCGAGTGCGTGCAGTCAAGTTTCGCATCATACTTGCGGAACAGGGTTATGACCTGCAGACGCTAAA
exu_MR   ACTACAAACTCAAAACGAGAATTTGGAGCGTCAAAACTCTTTCCGTCCGGTTTTCTTAAATTACTTTAAAACCACACTCTACCATCGAGTGCGTGCAGTCAAGTTTCGCATCATACTTGCGGAACAGGGTTATGACCTGCAGACGCTAAA

         ....|....|....|....|....|....|....|....|....|....|....|....|....|....|....|....|....|....|....|....|....|....|....|....|....|....|....|....|....|....|
             7355      7365      7375      7385      7395      7405      7415      7425      7435      7445      7455      7465      7475      7485      7495
exu_Gene TGCAATTTGGAGTGAGAAACAAAAGGATGGATTGGCCGAAGTATTGCAAGTCATTGAGGAGCTAAAACCTGAAGATAGGACGGAGTTGGCTGATTTATTTGACAGTTACTTCGATCCTGTAAAGACGACTATTAAGCCGATTGTCAAAAA
exu_FH   TGCAATTTGGAGTGAGAAACAAAAGGATGGATTGGCCGAAGTATTGCAAGTCATTGAGGAGCTAAAACCTGAAGATAGGACGGAGTTGGCTGATTTATTTGACAGTTACTTCGATCCTGTAAAGACGACTATTAAGCCGATTGTCAAAAA
exu_MH   TGCAATTTGGAGTGAGAAACAAAAGGATGGATTGGCCGAAGTATTGCAAGTCATTGAGGAGCTAAAACCTGAAGATAGGACGGAGTTGGCTGATTTATTTGACAGTTACTTCGATCCTGTAAAGACGACTATTAAGCCGATTGTCAAAAA
exu_FR   TGCAATTTGGAGTGAGAAACAAAAGGATGGATTGGCCGAAGTATTGCAAGTCATTGAGGAGCTAAAACCTGAAGATAGGACGGAGTTGGCTGATTTATTTGACAGTTACTTCGATCCTGTAAAGACGACTATTAAGCCGATTGTCAAAAA
exu_MR   TGCAATTTGGAGTGAGAAACAAAAGGATGGATTGGCCGAAGTATTGCAAGTCATTGAGGAGCTAAAACCTGAAGATAGGACGGAGTTGGCTGATTTATTTGACAGTTACTTCGATCCTGTAAAGACGACTATTAAGCCGATTGTCAAAAA

         ....|....|....|....|....|....|....|....|....|....|....|....|....|....|....|....|....|....|....|....|....|....|....|....|....|....|....|....|....|....|
             7505      7515      7525      7535      7545      7555      7565      7575      7585      7595      7605      7615      7625      7635      7645
exu_Gene CAACAATCGCCGCCGAAATAGACGCGGAGGTAGTGCAACATGCATGCATTAAGTTAAAGTGTATTGAAAAGCAATTAATCATATGATTTTCATTTTTTCTTTTTATTACTTAGTACGCAACATGAAGAACGACGGCAGCAATAGCGCTCG
exu_FH   CAACAATCGCCGCCGAAATAGACGCGGAG------------------------------------------------------------------------------------TACGCAACATGAAGAACGACGGCAGCAATAGCGCTCG
exu_MH   CAACAATCGCCGCCGAAATAGACGCGGAG------------------------------------------------------------------------------------TACGCAACATGAAGAACGACGGCAGCAATAGCGCTCG
exu_FR   CAACAATCGCCGCCGAAATAGACGCGGAG------------------------------------------------------------------------------------TACGCAACATGAAGAACGACGGCAGCAATAGCGCTCG
exu_MR   CAACAATCGCCGCCGAAATAGACGCGGAG------------------------------------------------------------------------------------TACGCAACATGAAGAACGACGGCAGCAATAGCGCTCG

         ....|....|....|....|....|....|....|....|....|....|....|....|....|....|....|....|....|....|....|....|....|....|....|....|....|....|....|....|....|....|
             7655      7665      7675      7685      7695      7705      7715      7725      7735      7745      7755      7765      7775      7785      7795
exu_Gene CACAAGTAGTGAGCAAGGAGCTGGCGGTGACAAGCAAGTCACTGGCGGTCAGCTGCCCGACACCACAATTAAGTCGCCATCTCCGCCAAACGGCGGCAAGCCGCAGCGTGGCAAGCGTAATCCGCGCTCACGCAATAACTCCTTAAAAAC
exu_FH   CACAAGTAGTGAGCAAGGAGCTGGCGGTGACAAGCAAGTCACTGGCGGTCAGCTGCCCGACACCACAATTAAGTCGCCATCTCCGCCAAACGGCGGCAAGCCGCAGCGTGGCAAGCGTAATCCGCGCTCACGCAATAACTCCTTAAAAAC
exu_MH   CACAAGTAGTGAGCAAGGAGCTGGCGGTGACAAGCAAGTCACTGGCGGTCAGCTGCCCGACACCACAATTAAGTCGCCATCTCCGCCAAACGGCGGCAAGCCGCAGCGTGGCAAGCGTAATCCGCGCTCACGCAATAACTCCTTAAAAAC
exu_FR   CACAAGTAGTGAGCAAGGAGCTGGCGGTGACAAGCAAGTCACTGGCGGTCAGCTGCCCGACACCACAATTAAGTCGCCATCTCCGCCAAACGGCGGCAAGCCGCAGCGTGGCAAGCGTAATCCGCGCTCACGCAATAACTCCTTAAAAAC
exu_MR   CACAAGTAGTGAGCAAGGAGCTGGCGGTGACAAGCAAGTCACTGGCGGTCAGCTGCCCGACACCACAATTAAGTCGCCATCTCCGCCAAACGGCGGCAAGCCGCAGCGTGGCAAGCGTAATCCGCGCTCACGCAATAACTCCTTAAAAAC
```

```
          ....|....|....|....|....|....|....|....|....|....|....|....|....|....|....|....|....|....|....|....|....|....|....|....|....|....|....|....|....|....|
              7805      7815      7825      7835      7845      7855      7865      7875      7885      7895      7905      7915      7925      7935      7945
exu_Gene  ACAGCTGCCAAGCGCCGCCGTCGACACGAGCAATACAACGGCAACAGCAACCAATTAAAAACTAAGAAGTTCATTCTAAGATTCAAGATAGGCGCTGTTGCGACATAAAAAAAACAAGTAATTTGTTTTATTTTTTTTTGTAATAGTTTT
exu_FH    ACAGCTGCCAAGCGCCGCCGTCGACACGAGCAATACAACGGCAACAGCAACCAATTAAAAACTAAGAAGTTCATTCTAAGATTCAAGATAGGCGCTGTTGCGACATAAAAAAAACAAGTAATTTGTTTTATTTTTTTTTGTAATAGTTTT
exu_MH    ACAGCTGCCAAGCGCCGCCGTCGACACGAGCAATACAACGGCAACAGCAACCAATTAAAAACTAAGAAGTTCATTCTAAGATTCAAGATAGGCGCTGTTGCGACATAAAAAAAACAAGTAATTTGTTTTATTTTTTTTTGTAATAGTTTT
exu_FR    ACAGCTGCCAAGCGCCGCCGTCGACACGAGCAATACAACGGCAACAGCAACCAATTAAAAACTAAGAAGTTCATTCTAAGATTCAAGATAGGCGCTGTTGCGACATAAAAAAAACAAGTAATTTGTTTTATTTTTTTTTGTAATAGTTTT
exu_MR    ACAGCTGCCAAGCGCCGCCGTCGACACGAGCAATACAACGGCAACAGCAACCAATTAAAAACTAAGAAGTTCATTCTAAGATTCAAGATAGGCGCTGTTGCGACATAAAAAAAACAAGTAATTTGTTTTATTTTTTTTTGTAATAGTTTT

          ....|....|....|....|....|....|....|....|....|....|....|....|....|....|....|....|....|....|....|....|....|....|....|....|....|....|....|....|....|....|
              7955      7965      7975      7985      7995      8005      8015      8025      8035      8045      8055      8065      8075      8085      8095
exu_Gene  CACCAACGTAGACACACTTTAGAGAGACGCCAGAAAAAAATTCTGGTATGCAAGTTGAATAGAATTTGAATTTTTAATTTTATATATGCCACATATTTTTATTATAATTATTATTACATGTATTGATTAGCTAAACGTACATGCATATAAT
exu_FH    CACCAACGTAGACACACTTTAGAGAGACGCCAGAAAAAAATTCTGGTATGCAAGTTGAATAGAATTTGAATTTTTAATTTTATATATGCCACATATTTTTATTATAATTATTATTACATGTATTGATTAGCTAAACGTACATGCATATAAT
exu_MH    CACCAACGTAGACACACTTTAGAGAGACGCCAGAAAAAAATTCTG---------------------------------------------------------------------------------------------------------
exu_FR    CACCAACGTAGACACACTTTAGAGAGACGCCAGAAAAAAATTCTGGTATGCAAGTTGAATAGAATTTGAATTTTTAATTTTATATATGCCACATATTTTTATTATAATTATTATTACATGTATTGATTAGCTAAACGTACATGCATATAAT
exu_MR    CACCAACGTAGACACACTTTAGAGAGACGCCAGAAAAAAATTCTG---------------------------------------------------------------------------------------------------------

          ....|....|....|....|....|....|....|....|....|....|....|....|....|....|....|....|....|....|....|....|....|....|....|....|....|....|....|....|....|....|
              8105      8115      8125      8135      8145      8155      8165      8175      8185      8195      8205      8215      8225      8235      8245
exu_Gene  TAACAAAAGCAAAAGAAATATGTATATATATATGTACACTTATTGAACGTTAGCAGTTACAATACCCAATTGTAGATGACGTTAGTTTAGAAAATATCTGATTATTGAAGTCATTAATAATTATTATTTTATCTGCTTTGCTACGCTGCA
exu_FH    TAACAAAAGCAAAAGAAATATGTATATATATATGTACACTTATTGAACGTTAGCAGTTACAATACCCAATTGTAGATGACGTTAGTTTAGAAAATATCTGATTATTGAAGTCATTAATAATTATTATTTTATCTGCTTTGCTACGCTGCA
exu_MH    ------------------------------------------------------------------------------------------------------------------------------------------------------
exu_FR    TAACAAAAGCAAAAGAAATATGTATATATATATGTACACTTATTGAACGTTAGCAGTTACAATACCCAATTGTAGATGACGTTAGTTTAGAAAATATCTGATTATTGAAGTCATTAATAATTATTATTTTATCTGCTTTGCTACGCTGCA
exu_MR    ------------------------------------------------------------------------------------------------------------------------------------------------------

          ....|....|....|....|....|....|....|....|....|....|....|....|....|....|....|....|....|....|....|....|....|....|....|....|....|....|....|....|....|....|
              8255      8265      8275      8285      8295      8305      8315      8325      8335      8345      8355      8365      8375      8385      8395
exu_Gene  AAGTACAACAACATAAATTAGTTTGCCAGCCGCAGGCAGAGGTGTAAGCTTTTCATAATTTATTTCGTAGTTTTACTAGAGTTGAGTTAAATAAAATGTAACAAATATTAGCTAACCTCTGGTTTGACGTCTTAGAGGATTCTACAAGAA
exu_FH    AAGTACAACAACATAAATTAGTTTGCCAGCCGCAGGCAGAGGTGTAAGCTTTTCATAATTTATTTCGTAGTTTTACTAGAGTTGAGTTAAATAAAATGTAACAAATATTAGCTAACCTCTGGTTTGACGTCTTAGAGGATTCTACAAGAA
exu_MH    ------------------------------------------------------------------------------------------------------------------------------------------------------
exu_FR    AAGTACAACAACATAAATTAGTTTGCCAGCCGCAGGCAGAGGTGTAAGCTTTTCATAATTTATTTCGTAGTTTTACTAGAGTTGAGTTAAATAAAATGTAACAAATATTAGCTAACCTCTGGTTTGACGTCTTAGAGGATTCTACAAGAA
exu_MR    ------------------------------------------------------------------------------------------------------------------------------------------------------
```

```
          ....|....|....|....|....|....|....|....|....|....|....|....|....|....|....|....|....|....|....|....|....|....|....|....|....|....|....|....|....|....|
             8405      8415      8425      8435      8445      8455      8465      8475      8485      8495      8505      8515      8525      8535      8545
exu_Gene  TTTGACAACCTTGCTTATTAACGCGTATTGAAATTTTGAATTTTTAGAAGGTTGCGCTTTAATATTATATTTGTTTTTTATGATTCCTATTATGATATTTATGTATTTATGCTATTTTGAAATTTTTGTTTAAAAAGAATACACAAGAAA
exu_FH    TTTGACAACCTTGCTTATTAACGCGTATTGAAATTTTGAATTTTTAGAAGGTTGCGCTTTAATATTATATTTGTTTTTTATGATTCCTATTATGATATTTATGTATTTATGCTATTTTGAAATTTTTGTTTAAAAAGAATACACAAGAAA
exu_MH    ------------------------------------------------------------------------------------------------------------------------------------------------------
exu_FR    TTTGACAACCTTGCTTATTAACGCGTATTGAAATTTTGAATTTTTAGAAGGTTGCGCTTTAATATTATATTTGTTTTTTATGATTCCTATTATGATATTTATGTATTTATGCTATTTTGAAATTTTTGTTTAAAAAGAATACACAAGAAA
exu_MR    ------------------------------------------------------------------------------------------------------------------------------------------------------

          ....|....|....|....|....|....|....|....|....|....|....|....|....|....|....|....|....|....|....|....|....|....|....|....|....|....|....|....|....|....|
             8555      8565      8575      8585      8595      8605      8615      8625      8635      8645      8655      8665      8675      8685      8695
exu_Gene  TATATAAACAAATATTTTTTTCTAACTTAAAAAAAAAAXXXXXXXXXXXXXXXXXXXCTCAAATTTAAAGCGAAAAAACCCCACAGTTTAGCTTCACAAGCAAAACACAAAAATTGATTGGTGCGTTGCAGTTATACATACATAGCAAAA
exu_FH    TATATAAACAAATATTTTTTTCTAACTTAAAAAAAAAA................................................................................................................
exu_MH    ---------------------------------------------------------CTCAAATTTAAAGCGAAAAAACCCCACAGTTTAGCTTCACAAGCAAAACACAAAAATTGATTGGTGCGTTGCAGTTATACATACATAGCAAAA
exu_FR    TATATAAACAAATATTTTTTTCTAACTTAAAAAAAAAA................................................................................................................
exu_MR    ---------------------------------------------------------CTCAAATTTAAAGCGAAAAAACCCCACAGTTTAGCTTCACAAGCAAAACACAAAAATTGATTGGTGCGTTGCAGTTATACATACATAGCAAAA

          ....|....|....|
             8705      8715
exu_Gene  CGAAAAAAAAAAAAA
exu_FH    ...............
exu_MH    CGAAAAAAAAAAAAA
exu_FR    ...............
exu_MR    CGAAAAAAAAAAAAA
```

Figure S1 Alignment of *exu* gene structure (exu_Gene) and the four alternative transcripts from *Anastrepha fraterculus* (exu_FH: Female head; exu_MH: Male head; exu_FR: Female reproductive; exu_MR: Male reproductive). The sequence of “N”s between 3925-5678 corresponds to a fraction of a large intron (~3.5 Kb) that was only partly sequenced, but its length was aproximately determined by PCR fragment size. A short sequence of “X”s (8589-8607) was included to account for a putative intron between the 3’UTR male exon and the rest of the *exu* sequence, since this last exon was only recovered by RACE PCR (as it is from processed cDNA, it does not include introns).

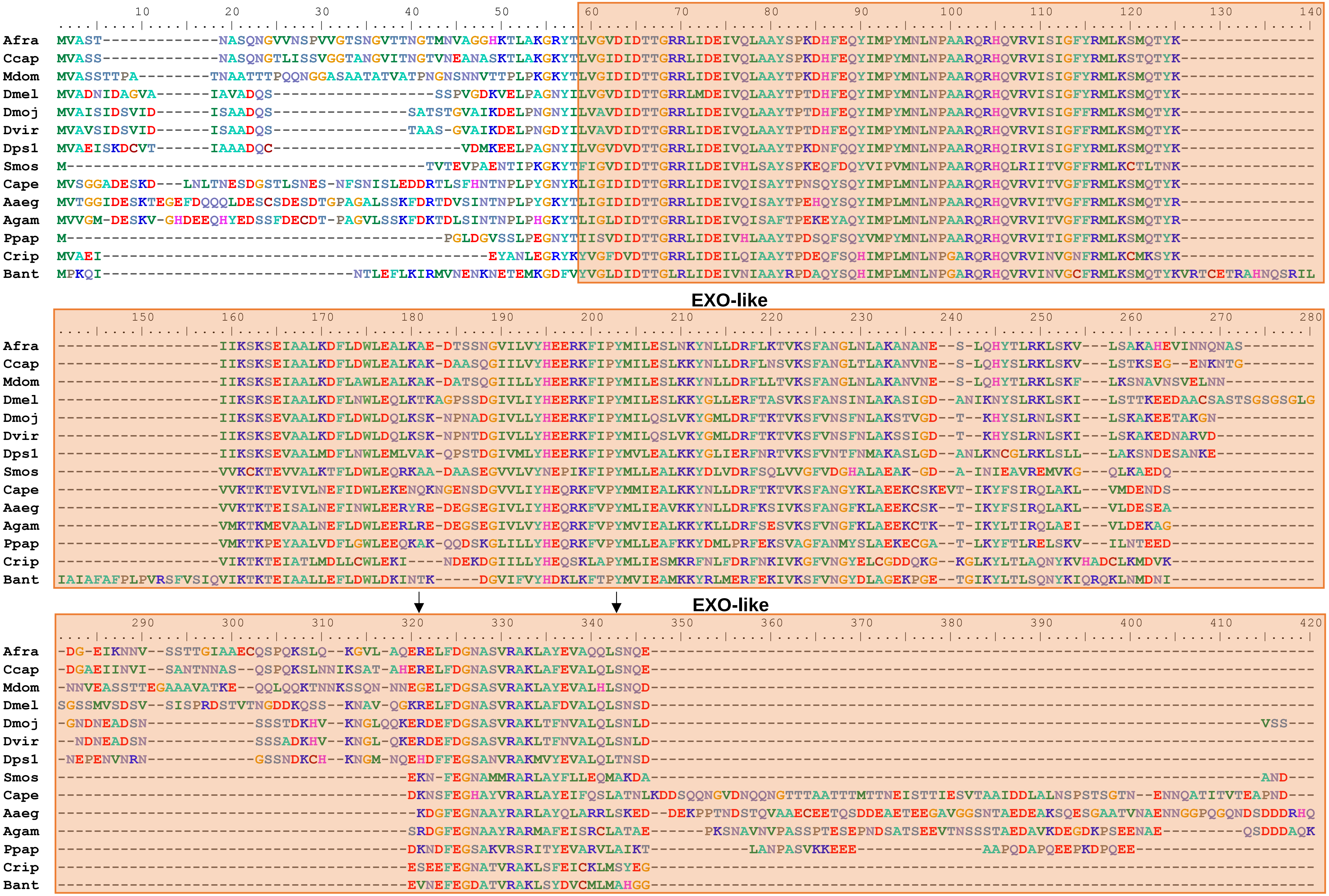
EXO-like
EXO-like

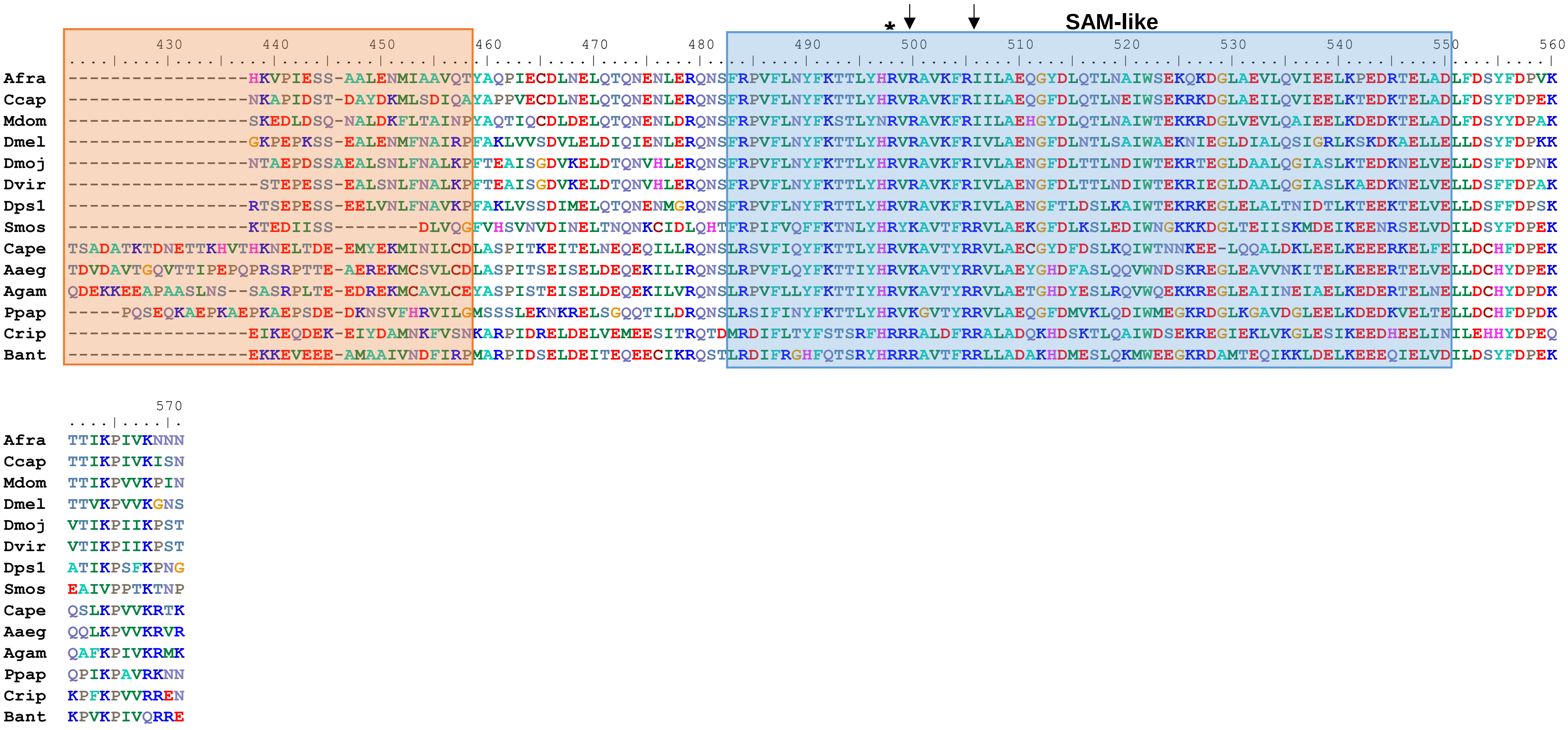

Figure S2 Alignment of the predicted amino acid sequences correspondent to the *exu* region (codons) used for evolutionary analyzes. Arrows indicate the four positions evolving under positive selection, and asterisk highlight the conserved Arg residue within the SAM-like domain. Afra: *Anastrepha fraterculus*; Ccap: *Ceratitis capitata*; Mdom: *Musca domestica*; Dmel: *Drosophila melanogaster*; Dmoj: *Drosophila mojavensis*; Dvir: *Drosophila virilis*; Dps1: *Drosophila pseudoobscura*; Smos: *Sitodiplosis mosellana*; Cape: *Corethrella appendiculata*; Aaeg: *Aedes aegypti*; Agam: *Anopheles gambiae*; Ppap: *Phlebotomus papatasi*; Crip: *Chironomus riparius*; and Bant: *Belgica antartica*

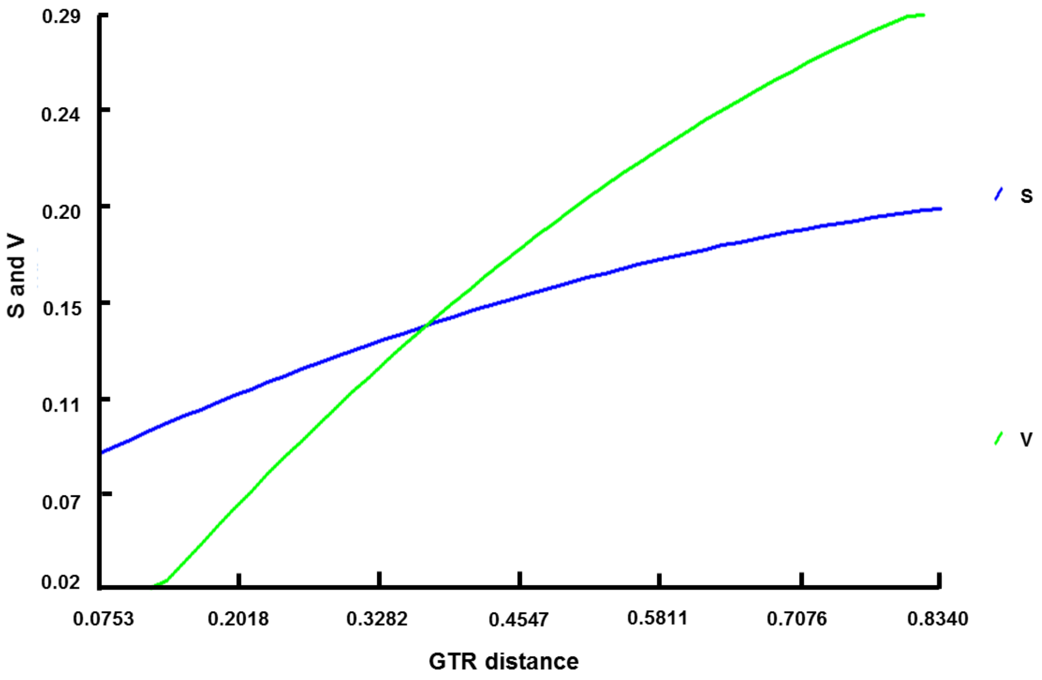

Figure S3 Substitution rates by genetic distance. Transition (s) and transversion (v) rates at *exu* against GTR distances

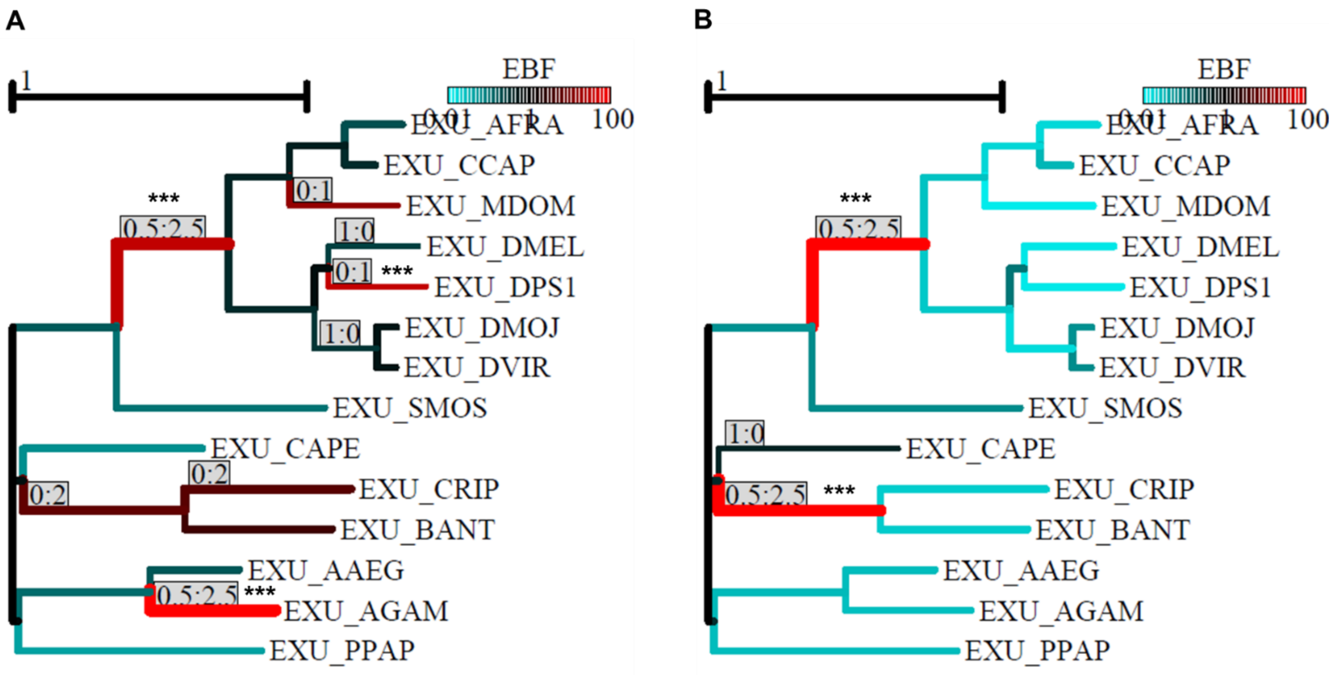

Figure S4 Episodes of diversifying selection in *exu* including the Cyclorrhapha branch. Among the four sites evolving under positive selection identified by MEME, two occur at the Cyclorrhapha branch: codons 321 (A) and 500 (B). Branches with evidence of positive selection ($P > 0.95$) are highlighted by asterisks. Numbers above each branch correspond to Synonymous:Nonsynonymous substitution counts. Afra: *Anastrepha fraterculus*; Ccap: *Ceratitis capitata*; Mdom: *Musca domestica*; Dmel: *Drosophila melanogaster*; Dmoj: *Drosophila mojavensis*; Dvir: *Drosophila virilis*; Dps1: *Drosophila pseudoobscura*; Smos: *Sitodiplosis mosellana*; Cape: *Corethrella appendiculata*; Aaeg: *Aedes aegypti*; Agam: *Anopheles gambiae*; Ppap: *Phlebotomus papatasi*; Crip: *Chironomus riparius*; and Bant: *Belgica antartica*

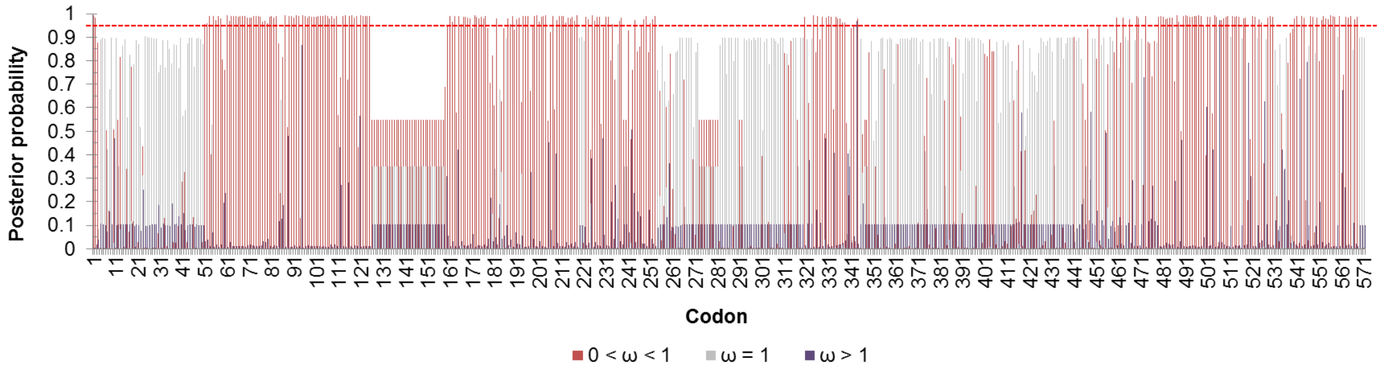

Figure S5 Evolutionary patterns of *exu* in Cyclorrhapha. Bayes Empirical Bayes posterior probabilities of codons under purifying selection (0 < ω < 1), evolving neutrally (ω = 1) and under positive selection (ω > 1). The two codons under positive selection (343 and 506) are highlighted by arrows, and refer to positions at the alignment. The dashed-line represents a threshold of *P* = 0.95

Table S1 RELAX results for selection relaxation or intensification in Tephritidae (test branch) compared to other Cyclorrhapha (reference branch), showing that there is no significant difference in selection strength between these two groups (*P* > 0.05)

Model	*k*	*log* L	*LRT*	*P*
Null	13.78	-14886.95	1.36	0.24
Alternative		-14886.27		
